# School phone policies and their association with mental wellbeing, phone use, and social media use (SMART Schools): a cross-sectional observational study

**DOI:** 10.1016/j.lanepe.2025.101211

**Published:** 2025-02-04

**Authors:** Victoria A. Goodyear, Amie Randhawa, Péymane Adab, Hareth Al-Janabi, Sally Fenton, Kirsty Jones, Maria Michail, Breanna Morrison, Paul Patterson, Jonathan Quinlan, Alice Sitch, Rebecca Twardochleb, Matthew Wade, Miranda Pallan

**Affiliations:** a School of Sport, Exercise and Rehabilitation Sciences, University of Birmingham, Birmingham, UK; b Institute for Mental Health, University of Birmingham, Birmingham, UK; c Department of Applied Health Sciences, University of Birmingham, Birmingham, UK; d National Institute for Health and Care Research (NIHR) Birmingham Biomedical Research Centre, UK; e Services for Education, Birmingham, UK; f School of Psychology, University of Birmingham, Birmingham, UK; g Forward Thinking Birmingham, Birmingham Women's and Children's NHS Foundation Trust, Birmingham, UK; h ukactive, ukactive Research Institute, London, UK; i Advanced Wellbeing Research Centre, College of Health, Wellbeing and Life Sciences, Sheffield Hallam University, Sheffield, UK

**Keywords:** Mobile phones, School policies, Mental health, Public health

## Abstract

**Background:**

Poor mental health in adolescents can negatively affect sleep, physical activity and academic performance, and is attributed by some to increasing mobile phone use. Many countries have introduced policies to restrict phone use in schools to improve health and educational outcomes. The SMART Schools study evaluated the impact of school phone policies by comparing outcomes in adolescents who attended schools that restrict and permit phone use.

**Methods:**

We conducted a cross-sectional observational study with adolescents from 30 English secondary schools, comprising 20 with restrictive (recreational phone use is not permitted) and 10 with permissive (recreational phone use is permitted) policies. The primary outcome was mental wellbeing (assessed using Warwick–Edinburgh Mental Well-Being Scale [WEMWBS]). Secondary outcomes included smartphone and social media time. Mixed effects linear regression models were used to explore associations between school phone policy and participant outcomes, and between phone and social media use time and participant outcomes. Study registration: ISRCTN77948572.

**Findings:**

We recruited 1227 participants (age 12–15) across 30 schools. Mean WEMWBS score was 47 (SD = 9) with no evidence of a difference between groups (adjusted mean difference −0.48, 95% CI −2.05 to 1.06, p = 0.62). Adolescents attending schools with restrictive, compared to permissive policies had lower phone (adjusted mean difference −0.67 h, 95% CI −0.92 to −0.43, p = 0.00024) and social media time (adjusted mean difference −0.54 h, 95% CI −0.74 to −0.36, p = 0.00018) during school time, but there was no evidence for differences when comparing usage time on weekdays or weekends.

**Interpretation:**

There is no evidence that restrictive school policies are associated with overall phone and social media use or better mental wellbeing in adolescents. The findings do not provide evidence to support the use of school policies that prohibit phone use during the school day in their current form, and indicate that these policies require further development.

**Funding:**

Public Health Research Programme, 10.13039/501100000272National Institute for Health and Care Research, Department of Health and Social Care, UK.

**Research in context**

**Evidence before this study**

Previous literature mainly reports negative associations between phone and social media use and mental health and wellbeing. This topic has been a prominent focus for research, but the evidence is currently weak, mixed, and largely based on ecological studies, and is consequently insufficient to inform policy and clinical practice. School phone policies that restrict the daytime use of phones are purported as an intervention to improve adolescent mental wellbeing and academic outcomes.

In March 2022, we conducted a literature search on EBSCO, Google Scholar, and Pub Med to identify studies published in English, up to March 2022, that explored the relationship between school phone policies and mental health and wellbeing, academic performance, and related outcomes (e.g., sleep and physical activity). Search terms included: smartphones or mobile phones or social media; school phone bans or phone limits or phone restrictions; adolescents or children or young people; mental health or mental wellbeing or anxiety or depression; physical activity or sedentary behaviour; sleep; attainment or achievement; behaviour or disruption; addictive or problematic use. We updated this search in February 2024. We found systematic reviews on the associations between screen-based activities and mental health outcomes, but we did not identify any published, peer-reviewed studies reporting on the influence of school phone policies on mental health and wellbeing. The SCAMP Study provides some evidence that restrictive school phone policies in the UK lower adolescents’ uses of electronic communication devices (including mobile phones), but not on health-related outcomes. There are a small number of grey literature articles reporting on school phone policies that restrict the daytime use of phones and their influence on mental wellbeing and academic performance in Europe. However, these latter articles are not peer-reviewed, and most of the conclusions have been drawn from administrative and ecological data collected prior to 2018, and/or from small scale non-representative studies that report descriptive data collected from non-validated measures.

**Added value of this study**

Our study is the first evaluation of school phone policies on mental health and wellbeing and other health and education-related outcomes which involves a nationally representative sample, and that reports associations between phone/social media use and health and educational outcomes using individual-level data in a large cohort of adolescents. Comparing schools that restrict the daytime use of phones with schools that permit it, we observed no differences in adolescents’ self-reported mental wellbeing, anxiety, depression, problematic social media use, and their motives for using social media. In addition, adolescents attending schools with restrictive phone policies did not differ in their sleep duration and efficiency, physical activity, academic attainment, and disruptive classroom behaviour, compared to pupils who attended schools where phone use was permitted during the school day. One potential explanation for this lack of observed difference, is that restrictive school phone policies did not lower the overall time adolescents spent on their phones/social media. Importantly our data also provide evidence to support claims of adverse consequences associated with increased overall phone/social media use. We observed that increased time spent on phones/social media is significantly associated with worsened outcomes for mental health and wellbeing, physical activity and sleep, and attainment and disruptive behaviour.

**Implications of all the available evidence**

This study does not provide evidence to support the use of school policies that prohibit phone use during the school day in their current form, given that no differences were observed in mental, physical, and academic outcomes for adolescents attending schools that permit, versus restrict phone use. However, the negative associations found between increasing time spent on phones/social media and worsened mental health and wellbeing do provide evidence on the need to address phone and social media use in adolescents, and school policies should be developed as part a more holistic approach. Preventative efforts should also consider how other behaviours that influence mental health and wellbeing are influenced by increased phone/media use, such as sleep, physical activity, attainment, classroom behaviour and problematic use.

## Introduction

The prevalence of adolescent mental health problems has increased exponentially in recent decades, especially in Western countries.^1^ In the UK, rates of probable mental disorders (mostly anxiety and depression) for those aged 8–16 rose from 12.5% in 2017 to 20.3% in 2023.^2^ Poor mental wellbeing negatively affects other aspects of adolescents’ lives, and is associated with higher rates of disruptive behaviour, school absence, lower educational attainment, sleep problems and sedentary lifestyles.^3^, ^4^, ^5^, ^6^, ^7^

In parallel, has been the constant increase in the ownership and use of smartphones and social media. These technologies have become integral aspects of adolescents’ lives.^8^ Studies from the UK report that adolescents spend on average 4 h per day on smartphones and up to 5 h on social media,^9^^,^^10^ and comparable trends have been reported across Europe^11^ and in the U.S.^12^ Problematic social media use is also common, with data from 29 countries showing prevalence rates ranging between 3 and 14% among adolescents.^13^

There is a body of research on associations between phone and/or social media time and adolescent health and wellbeing.^14^ This suggests that in moderation (e.g., <2 h per day) screen-based activities can be advantageous for mental health and wellbeing, as well as other associated health and behavioural outcomes (e.g., sleep, physical activity, classroom behaviour, and attainment).^14^, ^15^, ^16^ At higher levels of use the reverse effect tends to be seen, with increasing time spent on phones and social media associated with decreasing levels of mental wellbeing and higher levels of anxiety, and depression.^14^^,^^16^, ^17^, ^18^, ^19^, ^20^, ^21^, ^22^ Poor academic performance, disruptive classroom behaviour, and less time spent in physical activity and sleep are also more likely in adolescents who spend a greater proportion of time on smartphones and social media.^14^^,^^16^, ^17^, ^18^, ^19^^,^^21^^,^^23^ However, uncertainties in the strength of associations between smartphone/social media and mental wellbeing exist, and this is mainly due to how phone/social media use is measured and the predominance of ecological data to examine associations.^8^^,^^14^^,^^19^ Emerging evidence indicates that impacts of phone and social media use also require careful contextualisation, for factors, such as sex and age.^18^^,^^24^

In the last few years, there has been a growing international trend for the use of phones to be prohibited in schools.^25^ The UN reported that one in four countries (including France, Israel, and Turkey, as well as regions of Canada and Australia) have introduced laws that mandate public schools to prohibit phone use during the school day.^25^ Other countries, such as the UK, provide non-statutory guidance recommending prohibiting phones in schools, where prohibiting phone use is left to the school's discretion.^26^ However, prior to recent legislation and guidance, many schools had opted to devise their own policies that restrict phone use during the school day.^25^, ^26^, ^27^ Overall, restrictive school phone policies are based on popular assumptions that prohibiting phone use in schools will improve mental health and wellbeing, educational attainment, and reduce problematic use and levels of disruptive behaviour.^25^, ^26^, ^27^ There is some evidence indicating that restrictive school phone policies in the UK lower adolescents' uses of electronic communication devices (including mobile phones).^28^ However, there are currently no published peer-reviewed studies reporting on the association between school phone policies, adolescent phone/media use behaviours and mental health, wellbeing and other related outcomes (e.g., sleep, physical activity, educational attainment, and behaviour).^26^^,^^27^

Based on the available evidence of associations between phone use and mental health and wellbeing, we hypothesised that school policies that restrict the daytime use of phones would lower the overall time adolescents spend on phones/social media and improve adolescent mental wellbeing, possibly operating through improving related outcomes (e.g., physical activity, sleep, academic performance, and classroom behaviour). The logic model in Supplementary Figure S1 presents these processes. The research questions for this study were:

1. In schools that do not permit smartphone use compared with schools that permit smartphone use:
  a) Is there a difference in mental wellbeing, anxiety and depression, sleep duration, time spent in physical activity, classroom disruptive behaviour, attainment, and prevalence of problematic use?
  b) Is there a difference in smartphone and social media use and duration of use within school, over a 24 hr period and across 7 days, and is there a difference in motives for phone/media use?
2. Is there an association between smartphone and social media time and mental wellbeing, anxiety, depression, sleep duration, time spent in physical activity, classroom disruptive behaviour, attainment, and prevalence of problematic use?

## Methods

### Study design and participants

The SMART Schools study was a multi-method cross-sectional observational study, designed to evaluate the impact of school phone policies by comparing mental health and wellbeing, sleep, physical activity, and education outcomes in adolescents who attended schools that restrict and permit phone use during the school day. In this paper we focus on the findings from the quantitative observational element of the study.

This SMART Schools study exploited the natural variation in school phone policies across schools in England. We collected data in 30 secondary schools between November 2022 and November 2023. The study involved human participants and full ethical approval was obtained from the University of Birmingham's Science Technology, Engineering and Mathematics Research Ethics Committee on 8th July 2022 (ERN_22–0723). Full details of the SMART Schools study are described in the protocol.^27^ Our study followed the STROBE guidelines for reporting observational studies, see Supplementary File.

**Fig. 1:**
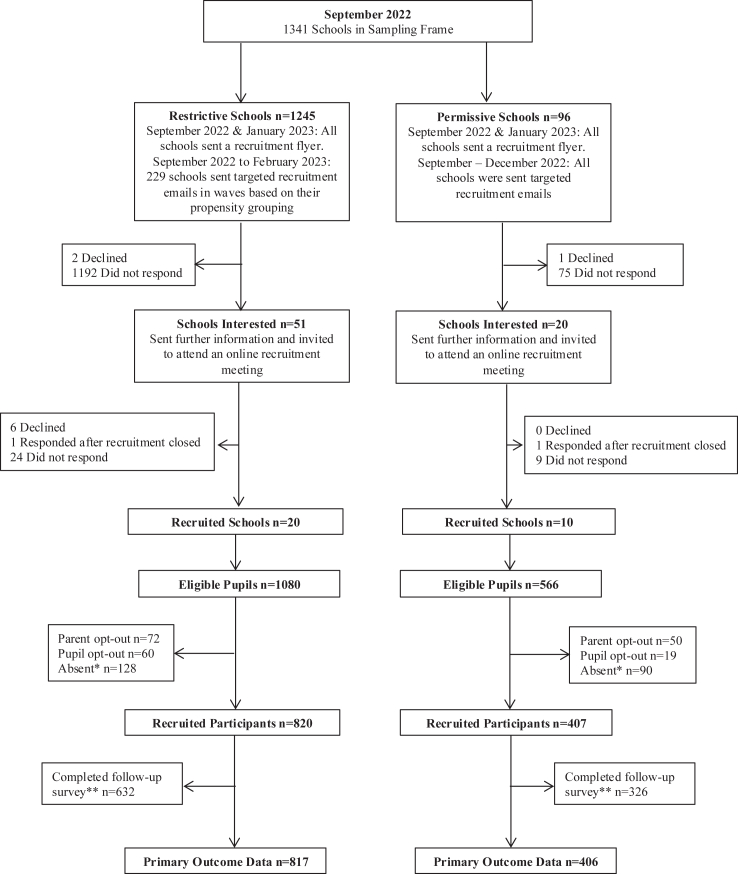
Study profile. Notes: ∗Absent includes pupils that were absent from school, out on school excursions, or in isolation/behavioural management. ∗∗Follow up surveys were not completed due to pupil absence, pupils’ declining, or school non-engagement.

School recruitment commenced in September 2022 and was completed in March 2023. The recruitment process is outlined in Fig. 1. The final sampling frame included 1341 state funded mainstream secondary schools (age 11–19) in England located within a 100-mile radius of the recruiting centre. Secondary schools were included as most adolescents in England own a smartphone by age 11.^9^ Schools other than state-funded mainstream schools (special schools, pupil referral units, and independent schools) were excluded because it was expected that there would be additional influences on mental wellbeing.^27^ Schools that did not have an accessible smartphone policy and/or had different phone policies for different year groups in mainstream education were excluded. Informed by our patient and public involvement (PPI) activities, school website, and policy analysis,^26^^,^^27^ we classified school phone policies as either restrictive (intervention) or permissive (comparator). In restrictive schools, phones were not allowed to be used during the school day for recreational purposes, and were required to be kept off inside bags, stored in lockers, kept in a pouch, handed into the school reception, or phones were not allowed onto the school premises altogether (see Table 1).^26^ In permissive schools, phones were permitted to be used at any time or at certain times (e.g., breaks/lunch) and/or in certain zones (e.g., outside) (see Table 1).^26^

**Table 1:** Different types of school phone policies within the study sample.

Policy categories	Number of schools adopting policy type	Sub-categories
**Permissive schools**		
1: Phones are allowed to be used at any time during the school day	1	N/A
2: Phones are allowed to be used at school during certain times/in certain areas	9	(2a) Phones are allowed during lunch and break times (7 schools)
		(2b) Phones are allowed to be used in designated zones (2 schools)
**Restrictive schools**		
3: Phones are not allowed to be used at school but are accessible to pupils	16	Phones must be kept off inside bags (16 schools)
4: Phones are not allowed to be used at school and are inaccessible to pupils	4	(4a) Phones must be kept in lockers (1 school)
		(4b) Phones must be kept in a pouch (1 school)
		(4c) Phones must be handed in to the school (1 school)
		(4d) Phones are not allowed on school premises (1 school)

To improve comparability of the two school policy groups, stratified sampling based on propensity scores^29^ was employed. We obtained routine data from the Department for Education on school characteristics: region, school type, urban or rural, total pupil roll size, Income Deprivation Affecting Children Index (IDACI), inclusion of a sixth form, selective or non-selective admissions policy, religious affiliation, and the proportion of pupils with the following characteristics: male, from minority ethnic groups, English as an additional language, eligible for free school meals, and special education needs. Propensity scores were calculated by regressing school phone policy type (restrictive or permissive) on these school characteristics. Propensity score terciles were then used to create three groups with subsequent division by restrictive or permissive policy type, resulting in six distinct sampling groups. Schools in each group were randomly ordered and invited sequentially to participate.

Data collection within recruited schools was completed between November 2022 and November 2023. School smartphone policies were identified using data collected from school policy documents that were accessed from school websites or from the school administration teams. The school policy category and type of permissive or restrictive policy (see Table 1) was verified during recruitment and consent meetings with school staff. In each school, a mixed-ability class of pupils in both year 8 (age 12–13) and year 10 (age 14–15) were selected. There were no ineligibility criteria for pupils, and all pupils within the participating classes were invited to take part, reducing any potential for selection bias. The form teacher for each class was recruited and asked to provide data on pupil attainment and behaviour. Pupils and teacher participants were asked to provide online or written consent (or assent), and parents/carers of pupil participants were given the opportunity to complete and return a written or online opt-out form. Our decision to use opt-out parental consent had the aim of reducing any socio-economic bias in the sample, further details of which are discussed elsewhere.^30^

### Procedures and outcomes

The primary outcome was mental wellbeing, measured using the Warwick–Edinburgh Mental Well-Being Scale (WEMWBS).^31^ WEMWBS has been validated for use in schools and has been used widely, nationally and internationally, for the evaluation of school projects and policies which aim to improve mental wellbeing.^31^ The items are all worded positively, covering both feeling and functioning aspects of mental wellbeing.^31^ As wellbeing fluctuates due to other factors (e.g., exam periods, school holidays, etc.), data on mental wellbeing were collected from each participating pupil at two time points. Secondary health and education-related outcomes assessed in pupil participants were: anxiety, depression, physical activity, sleep, classroom behaviour, attainment, and problematic social media use. Further secondary outcomes were smartphone and social media use duration and motives for social media use. Demographic variables (e.g., age, sex, socio-economic, ethnicity) were collected from each pupil.

Data collection methods included self-administered surveys for pupils and teachers (completed using university-approved online survey software (REDCap)), and accelerometer measured physical activity and sleep data for pupils. The pupil online survey was completed on university-owned encrypted tablets in the presence of researchers and a member of school staff and included validated measures for the primary and secondary outcomes, and motives for social media use. Smartphone and social media use duration were reported by pupils from their phones using iOS (Screentime) or Android (Digital Wellbeing) apps (self-reported [SR] phone data), and they self-reported time spent on their smartphone and social media (Supplementary File). Pupils were provided with a GENEActiv accelerometer watch, to be worn on the non-dominant wrist continuously for 24 h a day for the subsequent seven days after survey completion. Pupils were asked to wear the watch during all activities, including water-based activities. The second pupil survey measuring mental wellbeing only, was completed 4–8 weeks later, during school time and in the presence of school staff.

Data were collected from the form teacher for each participating pupil on their attainment and classroom behaviour, and whether they were eligible for free school meals (FSM), had a special educational need (SEN), or had English as an additional language (EAL). Teachers were emailed with the link to the REDCap survey to provide this data and were asked to complete the survey. Further details on the outcomes and the procedures for data collection are reported in the Supplementary File.

### Statistical analysis

To account for the imbalance of schools in our sampling frame that had restrictive (n = 1245) and permissive policies (n = 96), we recruited schools using a 2:1 ratio. The primary outcome of mental wellbeing was measured using WEMWBS (score range = 14–70). To detect a mean difference in score of 3 (considered the minimum clinically important difference^32^ between the two school groups), assuming a SD of 6.8^33^ and an ICC of 0.1 (a conservative estimate),^34^ with 90% power and 5% significance, we required 20 schools in the restrictive and 10 schools in the permissive phone policy groups, with an average cluster size of 39 pupils (1170 pupil participants in total; 780 in the restrictive, and 390 in the permissive policy groups).

The available data for each variable included in the analysis is reported in the Supplementary File. Missing data were not imputed, other than for the following: where a pupil had missing IMD (Index of Multiple Deprivation) data (n = 120), the median IMD rank for pupils in their school was used; where a pupil had a missing Date of Birth (n = 4), the median age for their year group in their school was used; missing responses on validated outcome measures were addressed as outlined in the Supplementary File.

**Table 2:** School and participant characteristics.

	Permissive schools	Restrictive schools	Statistical comparison test results
**School characteristics**	N = 10	N = 20	N = 30
**Region**			p = 0.12
East Midlands	1 (10.00%)	4 (20.00%)	
North West	2 (20.00%)	0 (0.00%)	
South East	3 (30.00%)	6 (30.00%)	
South West	3 (30.00%)	2 (10.00%)	
West Midlands	1 (10.00%)	8 (40.00%)	
**Urban location**	8 (80.00%)	17 (85.00%)	p > 0.99
**School size** median (IQR)	998 (693, 1186)	1120 (890, 1338)	p = 0.38
**IDACI deciles**			
1–5	6 (60.00%)	10 (50.00%)	p = 0.71
6–10	4 (40.00%)	10 (50.00%)	
**Religious affiliation: secular**	7 (70.00%)	18 (90.00%)	p = 0.30
**Admissions: selective**	3 (30.00%)	1 (5.00%)	p = 0.10
**School type: single sex**	2 (20.00%)	1 (5.00%)	p = 0.25
**% BME** mean (SD)	36 (28)	31 (26)	p = 0.64
**%FSM** mean (SD)	16 (11)	19 (8)	p = 0.47
**Has a sixth form**	7 (70.00%)	15 (75.00%)	p > 0.99
**Student responses**	38 (36, 46)	41 (37, 46)	p = 0.79
**Participant characteristics**	**Permissive**, N = 407	**Restrictive**, N = 820	

**Summer participation**	205 (50.37%)	296 (36.10%)	p < 0.0001
**Year group**			
Year 8	189 (46.44%)	405 (49.39%)	p = 0.36
Year 10	218 (53.56%)	415 (50.61%)	
**Age** mean (SD)	14.24 (1.14)	13.97 (1.07)	p < 0.0001
**Sex**			p = 0.15
Female	186 (45.93%)	420 (51.79%)	
Male	214 (52.84%)	381 (46.98%)	p = 0.053
Prefer not to say	5 (1.23%)	10 (1.23%)	p = 0.83
Unknown	2 (0.5%)	9 (1.1%)	
**SEN–student level**	55 (13.78%)	89 (12.13%)	p = 0.48
Unknown	8 (2.0%)	86 (10.0%)	
**EAL–student level**	85 (21.30%)	130 (17.71%)	p = 0.16
Unknown	8	86	
**FSM–student level**	65 (16.29%)	146 (19.89%)	p = 0.16
Unknown	8 (2.0%)	86 (10%)	
**Ethnicity**			p < 0.0001
White	263 (64.62%)	591 (72.07%)	
Mixed/Multiple ethnic groups	27 (6.63%)	38 (4.63%)	p = 0.072
Asian/Asian British	89 (21.87%)	103 (12.56%)	p < 0.0001
Black/African/Caribbean/Black British	12 (2.95%)	51 (6.22%)	p = 0.05
Other ethnic group/I would rather not say/Missing	16 (3.93%)	37 (4.51%)	p = 0.93

**Notes**: Data are n (%) unless otherwise stated. Inferential tests were done on the numerical variables (Kruskal–Wallis test for variables with medians listed and t-test for variables with means listed) and the binary categorical variables (Fisher's exact test for school characteristics and chi-square for participant characteristics) with the p values shown in the characteristics column. IDACI = Income Deprivation Affecting Children Index. Deciles 1 to 5 are those in the most deprived deciles, Deciles 6 to 10 are those in least deprived deciles. BME = Pupils from Black and Minority Ethnic Groups. FSM = Pupils with Special Educational Needs. SEN = Pupils with Special Educational Needs. EAL = Pupils English as an Additional Language. We controlled for the time of year when data collection was completed and summer participation refers to the summer months (May–July).

**Table 3:** Unadjusted and Adjusted Mean Differences in Outcomes by School Phone Policy (restrictive vs permissive phone policy group).

Outcome	N	Unadjusted mean difference (95% CI, p value)	Unstandardized adjusted mean difference (95% CI, p value)	Standardized adjusted mean difference (95% CI)
**Mental health and wellbeing**				
Mental wellbeing	1212	−1.62 (−3.32 to 0.07, p = 0.072)	−0.48 (−2.05 to 1.06, p = 0.62)	−0.05 (−0.23 to 0.12)
Anxiety	1206	0.65 (−0.21 to 1.51, p = 0.14)	0.10 (−0.76 to 0.97, p = 0.84)	0.02 (−0.14 to 0.18)
Depression	1198	0.67 (−0.30 to 1.65, p = 0.19)	−0.04 (−0.98 to 0.92, p = 0.94)	−0.01 (−0.16 to 0.15)
Problematic social media use	1190	3.61 (−0.28 to 7.52, p = 0.081)	0.5 (−2.6 to 3.53, p = 0.80)	0.02 (−0.12 to 0.17)
**Sleep and physical activity**				
Sleep duration (minutes)	758	5.1 (−4.42 to 14.64, p = 0.30)	1.27 (−8.67 to 10.12, p = 0.82)	0.03 (−0.19 to 0.22)
Sleep efficiency	758	0.14 (−1.01 to 1.28, p = 0.82)	0.40 (−0.83 to 1.60, p = 0.61)	0.08 (−0.16 to 0.31)
Sleep window	758	0.08 (−0.15 to 0.31, p = 0.51)	0.02 (−0.25 to 0.27, p = 0.92)	0.02 (−0.27 to 0.29)
Time of falling asleep	758	−0.01 (−0.30 to 0.28, p = 0.95)	−0.18 (−0.42 to 0.07, p = 0.21)	−0.17 (−0.40 to 0.07)
Wake up time	758	0.02 (−0.31 to 0.34, p = 0.92)	−0.22 (−0.56 to 0.12, p = 0.30)	−0.26 (−0.65 to 0.14)
Moderate-to-Vigorous PA	785	0.56 (−3.37 to 4.54, p = 0.78)	1.78 (−2.59 to 6.09, p = 0.46)	0.11 (−0.16 to 0.39)
Average daily acceleration	785	0.15 (−2.36 to 2.67, p = 0.91)	1.14 (−1.6 to 3.82, p = 0.45)	0.12 (−0.16 to 0.39)
**Attainment**				
Attainment English = below target^a^	1114	1.79 (0.94–3.40, p = 0.075)	1.45 (0.85–2.47, p = 0.18)	NA
Attainment Maths = below target^a^	1121	1.37 (0.63–3.00, p = 0.43)	1.01 (0.45–2.27, p = 0.98)	NA
**Classroom behaviour**				
Disruptiveness	1096	−0.12 (−0.7 to 0.46, p = 0.70)	0.06 (−0.57 to 0.68, p = 0.88)	0.03 (−0.25 to 0.30)
**Social media use motives**				
SMUM–conformity	1195	0 (−0.54 to 0.53, p = 0.99)	−0.24 (−0.81 to 0.30, p = 0.49)	−0.07 (−0.25 to 0.09)
SMUM–coping	1195	0.68 (0.09–1.28, p = 0.032)	0.1 (−0.53 to 0.72, p = 0.77)	0.02 (−0.12 to 0.17)
SMUM–enhancement	1194	0.06 (−0.64 to 0.76, p = 0.88)	−0.12 (−0.72 to 0.49, p = 0.74)	−0.03 (−0.18 to 0.12)
SMUM–social	1195	−0.33 (−1.09 to 0.43, p = 0.40)	−0.31 (−0.95 to 0.36, p = 0.45)	−0.07 (−0.21 to 0.08)
**Smartphone and social media use duration**				
Screen time–in school	1102	−0.47 (−0.72 to −0.22, p = 0.00094)	−0.67 (−0.92 to −0.43, p = 0.00024)	−0.47 (−0.65 to −0.3)
Screen time–weekday	1160	0.39 (−0.09 to 0.88, p = 0.12)	0.01 (−0.49 to 0.54, p = 0.96)	0.01 (−0.18 to 0.19)
Screen time–weekend day	1147	0.98 (0.12–1.83, p = 0.033)	0.58 (−0.32 to 1.50, p = 0.31)	0.15 (−0.08 to 0.39)
Screen time–week	1134	4.08 (−0.02 to 8.17, p = 0.062)	1.46 (−2.82 to 5.85, p = 0.59)	0.07 (−0.14 to 0.29)
Social media–in school	1102	−0.34 (−0.55 to −0.13, p = 0.0037)	−0.54 (−0.74 to −0.36, p = 0.00018)	−0.45 (−0.61 to −0.30)
Social media—weekday	1160	0.63 (0.24–1.02, p = 0.0043)	0.25 (−0.18 to 0.69, p = 0.33)	0.09 (−0.07 to 0.26)
Social media–weekend day	1147	1.13 (0.44–1.81, p = 0.0032)	0.61 (−0.12 to 1.36, p = 0.19)	0.17 (−0.03 to 0.38)
Social media–week	1134	5.50 (2.24–8.76, p = 0.0023)	2.66 (−0.67 to 6.08, p = 0.22)	0.14 (−0.04 to 0.32)

**Notes:** Wellbeing reported as WEMWBS score. Anxiety reported as GAD-7 score. Depression reported as PAQ-9 score. Problematic Social Media Use reported as PSMU score. Sleep Duration (hrs). Sleep Efficiency (%). Physical Activity (PA). Moderate-to-Vigorous PA (mins). Average Daily Acceleration (mg). Classroom Disruptiveness reported as PBQ score.

a Mixed effects logistic regression models; odd ratios reported for attainment below target vs attainment on or above target. Smartphone and Social Media Use Duration reported in hrs. School ID was included as a random effect in all models. Year group was included as a random effect in all models except for the unadjusted Attainment models. Fixed effects variables included in the adjusted models at the individual-level were: sex, and ethnicity, and at the school-level were: %Pupils with English as an Additional Language (EAL), %Pupils with Special Educational Needs (SEN), %Pupils eligible for (FSM), school size, school Income Deprivation Affecting Children Index (IDACI), school religious affiliation, school admissions policy, school co-education status, and month of measurement.

All analysis was conducted using R Statistics (version 4.1.2) and R Studio. For the primary outcome of mental wellbeing, a mean was calculated from the two measures for each individual and its association with phone policy type was investigated using mixed effects linear regression which included year group (year 8 or year 10) and school (30 schools) as random effects variables. School- and pupil-level covariates were included as fixed effects adjustment variables (see Supplementary File). The adjustment variables were identified based on the available literature on the key factors that may influence mental wellbeing and/or phone/media use.

The association between phone policy type and the secondary outcomes were explored using mixed effects linear regression, except for attainment scores, where mixed effects logistic regression was used. All regression models included the same random and fixed effects covariates except for the attainment mixed effects logistic regression model which included year group as a fixed, instead of a random effect to allow convergence. For all schools in the study, we investigated the association between smartphone and social media time and the primary and secondary outcomes using mixed effect models, including the same adjustment variables (Supplementary File).

Based on the literature on adolescent mental wellbeing, we identified four subgroup effects that were of interest: deprivation, sex, ethnicity, and year group. We separately introduced interactions between pupil sex, ethnicity, year group, school IDACI, and school smartphone policy into the models. We performed a sensitivity analysis using only the first WEMWBS score provided by participants to explore the potential influence of the experience of the first data collection point on the second completion of the WEMWBS questions. Self-reported phone use was used for our primary analysis due to concerns over the accuracy of the SR phone data (Supplementary File). There was a high proportion of missing data due to input errors and the way the phone apps may have been miscounting social time, resulting in nearly a third of participants reporting higher social media times than phone times. These issues were comparable across permissive and restrictive schools (see Supplementary File). We found that the self-reported and SR phone data measures had a strong correlation (Supplementary File). Only self-reported phone and social media use is reported from this point forwards. We modelled relationships between phone and social media time and other outcomes as linear based on our exploratory data analysis (Supplementary File), however we explored non-linearity in these relationships by re-running the models using a log (x + 1) transformation for the phone and social media duration variables (models are not reported as there were no meaningful changes in interpretation of the associations). Additionally, we performed a sensitivity analysis with a small sample using only data from pupils in schools where restrictive phone policies required phones to be inaccessible to pupils (n = 4), to explore whether policies with greater levels of restrictions on pupils’ access to phones influenced outcomes. We performed a sensitivity analysis with weekend smartphone use as the control variable to account for the relationship between in-school and leisure time phone use.

### Role of the funding source

This study is funded by the National Institute for Health Research (NIHR). The funder of the study had no role in study design, data collection, data analysis, data interpretation, or writing of the report, or the decision to submit the Article for publication.

## Results

325 schools were approached, and this included 229 restrictive schools and 96 permissive schools. The response rate at the school level was 8.73% for schools with restrictive phone policies and 10.42% for schools with permissive phone policies (see Fig. 1). The response rate at the pupil level was 75.93% for pupils in schools with restrictive policies, and 71.90% for pupils in schools with permissive policies (see Fig. 1). Of the recruited pupil participants, 1223 (99.67%) provided data on mental wellbeing (primary outcome); 817 (99.63%) in the restrictive schools (intervention) and 406 (99.75%) in permissive schools (comparator) (Fig. 1). Table 2 shows the characteristics of schools and pupil participants. The 30 participating schools did not differ considerably from the 1341 schools in the sampling frame across all characteristics (Supplementary File). The sample of recruited schools with a permissive school phone policy (comparator group) included a greater proportion of selective and single sex schools, compared to the restrictive schools (intervention), however, this difference was proportionately reflected in the sampling frame (Supplementary File). Pupil characteristics were well balanced across comparator and intervention schools (Table 2).

The majority of schools in the permissive school category permitted the use of phones at lunch/break and/or in certain zones, and the majority of schools in the restrictive category required phones to be kept off in school bags during the school day (see Table 1). The majority of restrictive and permissive phone policies were introduced in the last 2–5 years, although most restrictive policies were implemented in the last 1–2 years (see Supplementary File).

**Fig. 2:**
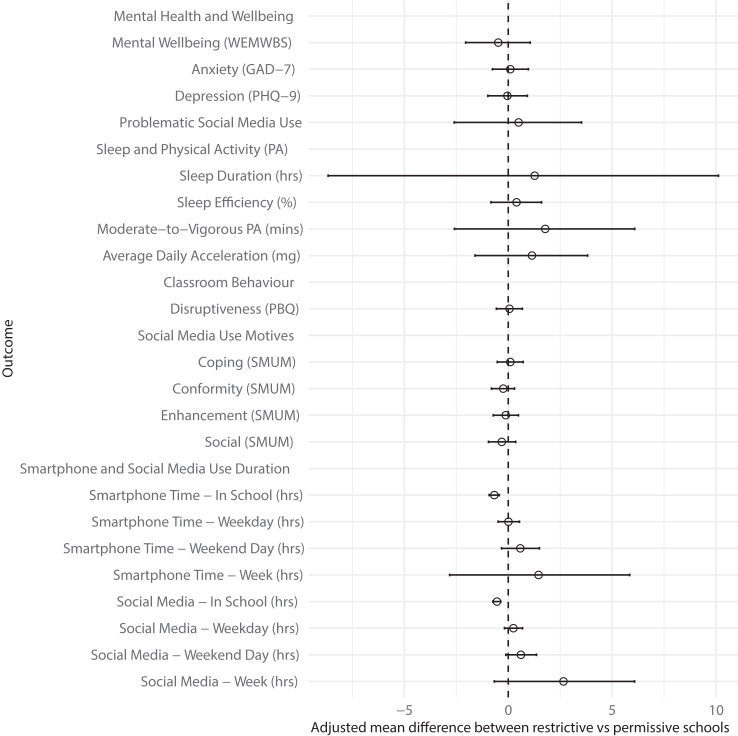
Adjusted mean differences in outcomes (mental health and wellbeing, sleep and physical activity, classroom behaviour and phone and social media use) by school phone policy.

The ICC for the primary outcome was 0.05. Mean WEMWBS mental wellbeing score was 47 (SD = 9) and was similar in the permissive (Mean = 48, SD = 9) and restrictive schools (Mean = 46, SD = 9), indicating a medium level of wellbeing across school groups,^26^ and with no evidence of a difference between groups when controlling for other variables (adjusted mean difference −0.48, 95% CI −2.05 to 1.06, p = 0.62, with permissive as reference group) (Fig. 2, Table 3). No statistically significant interactions with school policy were observed for mental wellbeing across sex, year group, ethnicity, and IDACI (see Supplementary File). In the sensitivity analysis using only the first WEMWBS measure there was no significant difference in mental wellbeing between the two school groups (see Supplementary File). No issues were found with excessive numbers of participants scoring 0 (n = 0) or maximum (n = 3) on the WEMWBS score.

Regarding the mental health outcomes of anxiety and depression, there was no evidence of a significant difference between permissive and restrictive schools (Fig. 2, Table 3). No significant differences in pupil outcomes were observed between permissive and restrictive schools for all other behavioural outcomes (Fig. 2, Table 3) or for attainment in English (adjusted odds ratio 1.45, 95% CI 0.85–2.47, p = 0.18, reference = permissive) and Maths (adjusted odds ratio 1.01, 95% CI 0.45–2.27, p = 0.98, reference = permissive).

The median smartphone time in school was 0.38 h (IQR 0.02–1.46) and was greater in permissive schools (1 h, IQR 0.50–2.00) compared to restrictive (0.17 h, IQR 0.00–1.00) (Supplementary File). Median social media time in school was 0.17 h (IQR 0.00–0.83) and was greater in permissive schools (0.50 h, IQR 0.17–1.17) compared to restrictive (0.03 h, IQR 0.00–0.50). Students attending restrictive schools had significantly lower in school smartphone time (adjusted mean difference −0.67 h, 95% CI −0.92 to −0.43, p = 0.00024) and social media time (adjusted mean difference −0.54 h, 95% CI −0.74 to −0.36, p = 0.00018) compared to students in permissive schools. There was some variability in the distribution of the time spent on smartphones and social media during school hours in both permissive and restrictive schools (Fig. 2). In the sensitivity analysis using only restrictive schools where phones were inaccessible to pupils, there were no significant differences with permissive schools across all outcomes (Supplementary File).

For all other measures of smartphone and social media time (on a weekday, on a weekend day, and over a week) the median use duration in restrictive schools was greater (Supplementary File), however in the adjusted models, the differences were non-significant (Fig. 2, Table 3). Almost all participants used their phones for >1.7 h on a weekday and >2 h a weekend day (Supplementary File). A weak positive correlation was found between smartphone time in school and smartphone time across the school day and weekend day (see Supplementary File). This weak positive correlation was also found between in-school social media time and social media time across the school day and on a weekend day (See Supplementary File). In the sensitivity analysis controlling for weekend smartphone time, no significant differences were found for all outcomes (see Supplementary File).

Increases in smartphone and social media time were associated with reduced mental wellbeing on a weekday, on a weekend day, and across a week, although in-school phone and social media use was non-significant (Table 4, Table 5). Increases in smartphone and social media time were associated with increases in anxiety, depression and problematic social media use across all measurement periods (Table 4, Table 5). Overall, this showed that an increase in smartphone and social media use time was related to worse mental health outcomes.

**Table 4:** Unadjusted and adjusted associations between smartphone time (measured in hours) and mental health, sleep, physical activity and educational outcomes: Mixed effects regression analyses.

Outcome	N	Unadjusted regression coefficient (95% CI)	Unstandardized adjusted regression coefficient (95% CI)	Standardized adjusted regression coefficient (95% CI)
**Associations for smartphone time—in school (hrs)**				
*Mental Wellbeing*	1101	−0.25 (−0.61 to 0.12, p = 0.18)	−0.25 (−0.58 to 0.12, p = 0.17)	−0.04 (−0.09 to 0.02)
Anxiety	1101	0.28 (0.06–0.50, p = 0.012)	0.30 (0.08–0.50, p = 0.0056)	0.08 (0.02–0.13)
Depression	1094	0.53 (0.27–0.78, p < 0.0001)	0.53 (0.27–0.76, p < 0.0001)	0.12 (0.06–0.18)
Problematic social media use	1096	1.64 (0.75–2.52, p = 0.00029)	1.63 (0.77–2.45, p = 0.00018)	0.11 (0.05–0.17)
*Sleep duration (minutes)*	701	−0.18 (−2.68 to 2.30, p = 0.88)	0.18 (−2.22 to 2.68, p = 0.89)	0.01 (−0.07 to 0.08)
*Sleep efficiency*	701	−0.24 (−0.51 to 0.04, p = 0.088)	−0.25 (−0.52 to 0.030, p = 0.080)	−0.07 (−0.14 to 0.01)
*Moderate-to-Vigorous PA*	723	−0.33 (−1.13 to 0.48, p = 0.43)	−0.38 (−1.16 to 0.41, p = 0.35)	−0.03 (−0.11 to 0.04)
*Average daily acceleration*	723	−0.26 (−0.77 to 0.25, p = 0.31)	−0.28 (−0.77 to 0.22, p = 0.27)	−0.04 (−0.11 to 0.03)
Disruptiveness	991	0.23 (0.14–0.32, p < 0.0001)	0.22 (0.13–0.31, p < 0.0001)	0.14 (0.08–0.20)
Attainment English = below target^a^	1001	1.14 (1.03–1.25, p = 0.011)	1.15 (1.04–1.27, p = 0.0058)	
Attainment Maths = below target^a^	1007	1.16 (1.05–1.28, p = 0.0039)	1.18 (1.06–1.30, p = 0.0017)	
**Associations for smartphone time –weekday (hrs)**				
*Mental wellbeing*	1159	−0.65 (−0.83 to −0.47, p < 0.0001)	−0.53 (−0.71 to −0.36, p < 0.0001)	−0.17 (−0.22 to −0.11)
Anxiety	1159	0.46 (0.35–0.57, p < 0.0001)	0.38 (0.28–0.48, p < 0.0001)	0.2 (0.14–0.25)
Depression	1153	0.62 (0.50–0.74, p < 0.0001)	0.54 (0.42–0.66, p < 0.0001)	0.25 (0.19–0.30)
Problematic social media use	1152	2.88 (2.48–3.29, p < 0.0001)	2.67 (2.26–3.06, p < 0.0001)	0.35 (0.30–0.40)
*Sleep duration (minutes)*	728	−2.77 (−4.02 to −1.51, p < 0.001)	−3.43 (−4.69 to −2.18, p < 0.0001)	−0.21 (−0.28 to −0.13)
*Sleep efficiency*	728	−0.13 (−0.27 to 0.01, p = 0.062)	−0.14 (−0.29 to 0.00, p = 0.053)	−0.08 (−0.15 to 0.00)
*Moderate-to-Vigorous PA*	751	−0.74 (−1.15 to −0.33, p = 0.00040)	−0.54 (−0.98 to −0.15, p = 0.011)	−0.1 (−0.17 to −0.03)
*Average daily acceleration*	751	−0.50 (−0.76 to −0.24, p = 0.00014)	−0.35 (−0.63 to −0.11, p = 0.0085)	−0.1 (−0.18 to −0.03)
Disruptiveness	1045	0.11 (0.07–0.16, p < 0.0001)	0.12 (0.08–0.17, p < 0.0001)	0.15 (0.10–0.21)
Attainment English = below target^a^	1054	1.10 (1.04–1.15, p = 0.00021)	1.10 (1.05–1.16, p = 0.00014)	
Attainment Maths = below target^a^	1061	1.11 (1.06–1.17, p < 0.0001)	1.10 (1.04–1.16, p = 0.00047)	
**Associations for smartphone time—weekend day (hrs)**				
*Mental wellbeing*	1146	−0.51 (−0.64 to −0.38, p < 0.0001)	−0.39 (−0.52 to −0.26, p < 0.0001)	−0.17 (−0.23 to −0.11)
Anxiety	1146	0.33 (0.25–0.41, p < 0.0001)	0.25 (0.17–0.33, p < 0.0001)	0.18 (0.12–0.24)
Depression	1140	0.45 (0.36–0.54, p < 0.0001)	0.38 (0.29–0.47, p < 0.0001)	0.24 (0.18–0.29)
Problematic social media use	1140	2.05 (1.75–2.35, p < 0.0001)	1.82 (1.51–2.11, p < 0.0001)	0.33 (0.28–0.39)
*Sleep duration (minutes)*	720	−1.56 (−2.42 to −0.68, p = 0.00039)	−2.01 (−2.85 to −1.11, p < 0.0001)	−0.17 (−0.24 to −0.09)
*Sleep efficiency*	720	−0.09 (−0.19 to 0.00, p = 0.058)	−0.11 (−0.20 to −0.01, p = 0.030)	−0.08 (−0.15 to 0.00)
*Moderate-to-Vigorous PA*	744	−0.52 (−0.80 to −0.24, p = 0.00026)	−0.35 (−0.65 to −0.09, p = 0.015)	−0.09 (−0.16 to −0.02)
*Average daily acceleration*	744	−0.37 (−0.55 to −0.20, p < 0.0001)	−0.26 (−0.44 to −0.09, p = 0.0046)	−0.10 (−0.17 to −0.03)
Disruptiveness	1033	0.05 (0.02–0.09, p = 0.0019)	0.06 (0.03–0.10, p = 0.00024)	0.11 (0.05–0.17)
Attainment English = below target^a^	1041	1.05 (1.01–1.08, p = 0.012)	1.06 (1.02–1.10, p = 0.0037)	
Attainment Maths = below target^a^	1048	1.06 (1.02–1.10, p = 0.0021)	1.05 (1.01–1.09, p = 0.013)	
**Associations for smartphone time—week (hrs)**				
*Mental wellbeing*	1133	−0.10 (−0.12 to −0.07, p < 0.0001)	−0.08 (−0.10 to −0.05, p < 0.0001)	−0.17 (−0.23 to −0.12)
Anxiety	1133	0.07 (0.05–0.08, p < 0.0001)	0.05 (0.04–0.07, p < 0.0001)	0.20 (0.14–0.26)
Depression	1127	0.09 (0.07–0.11, p < 0.0001)	0.08 (0.06–0.09, p < 0.0001)	0.26 (0.20–0.31)
Problematic social media use	1127	0.42 (0.37–0.48, p < 0.0001)	0.39 (0.33–0.44, p < 0.0001)	0.37 (0.31–0.42)
*Sleep duration (minutes)*	713	−0.39 (−0.56 to −0.22, p < 0.0001)	−0.49 (−0.66 to −0.32, p < 0.0001)	−0.22 (−0.29 to −0.14)
*Sleep efficiency*	713	−0.02 (−0.04 to 0.00, p = 0.044)	−0.02 (−0.04 to −0.00, p = 0.027)	−0.09 (−0.16 to −0.01)
*Moderate-to-Vigorous PA*	736	−0.11 (−0.16 to −0.05, p = 0.00013)	−0.08 (−0.14 to −0.02, p = 0.0081)	−0.10 (−0.18 to −0.03)
*Average daily acceleration*	736	−0.08 (−0.11 to −0.04, p < 0.001)	−0.05 (−0.09 to −0.02, p = 0.0043)	−0.11 (−0.18 to −0.04)
Disruptiveness	1022	0.01 (0.01–0.02, p < 0.001)	0.02 (0.01–0.02, p < 0.0001)	0.15 (0.09–0.21)
Attainment English = below target^a^	1030	1.01 (1.00–1.02, p = 0.0011)	1.01 (1.01–1.02, p = 0.00036)	
Attainment Maths = below target^a^	1037	1.01 (1.01–1.02, p = 0.0001)	1.01 (1.01–1.02, p = 0.0015)	

**Notes**: Wellbeing reported as WEMWBS score. Anxiety reported as GAD-7 score. Depression reported as PAQ-9 score. Problematic Social Media Use reported as PSMU score. Sleep Duration (hrs). Sleep Efficiency (%). Physical Activity (PA). Moderate-to-Vigorous PA (mins). Average Daily Acceleration (mg). Classroom Disruptiveness reported as PBQ score. Italics represent positive outcomes, regular text represents adverse outcomes.

a Mixed effects logistic regression models; odd ratios reported for attainment below target vs attainment on or above target. The exposure difference of coefficients is 1 h. School ID was included as a random effect in all models. N represents the N of the adjusted models. Year group was included as a random effect in all models except for the unadjusted Attainment models. Fixed effects variables included in the adjusted models at the individual-level were: sex, and ethnicity, and at the school-level were: %Pupils with English as an Additional Language (EAL), %Pupils with Special Educational Needs (SEN), %Pupils eligible for (FSM), school size, school Income Deprivation Affecting Children Index (IDACI), school religious affiliation, school admissions policy, school co-education status, and month of measurement.

**Table 5:** Unadjusted and adjusted associations between social media time (measured in hours) and mental health, sleep, physical activity and educational outcomes: Mixed effects regression analyses.

Outcome	N	Unadjusted regression coefficient (95% CI)	Unstandardized adjusted regression coefficient (95% CI)	Standardized adjusted regression coefficient (95% CI)
**Associations for social media time—in school (hrs)**				
*Mental wellbeing*	1101	−0.25 (−0.67 to 0.18, p = 0.25)	−0.22 (−0.61 to 0.21, p = 0.290)	−0.03 (−0.08 to 0.03)
Anxiety	1101	0.26 (0.00–0.51, p = 0.050)	0.26 (0.01–0.50, p = 0.040)	0.06 (0–0.11)
Depression	1094	0.57 (0.27–0.87, p = 0.00018)	0.56 (0.27–0.84, p = 0.00016)	0.11 (0.05–0.17)
Problematic social media use	1096	2.12 (1.08–3.16, p < 0.0001)	2.09 (1.10–3.07, p < 0.0001)	0.12 (0.06–0.18)
*Sleep duration (minutes)*	701	−1.07 (−4.05 to 1.92, p = 0.48)	−0.77 (−3.64 to 2.24, p = 0.61)	−0.02 (−0.10 to 0.06)
*Sleep efficiency*	701	−0.26 (−0.59 to 0.07, p = 0.13)	−0.28 (−0.62 to 0.04, p = 0.094)	−0.07 (−0.15 to 0.01)
*Moderate-to-Vigorous PA*	723	−0.34 (−1.31 to 0.63, p = 0.49)	−0.42 (−1.37 to 0.51, p = 0.40)	−0.03 (−0.11 to 0.04)
*Average Daily Acceleration*	723	−0.14 (−0.76 to 0.47, p = 0.65)	−0.18 (−0.78 to 0.41, p = 0.55)	−0.02 (−0.10 to 0.05)
Disruptiveness	991	0.31 (0.21–0.42, p < 0.0001)	0.31 (0.20–0.41, p < 0.0001)	0.17 (0.11–0.22)
Attainment English = below target^a^	1001	1.18 (1.05–1.32, p = 0.0046)	1.20 (1.07–1.35, p = 0.0019)	
Attainment Maths = below target^a^	1007	1.15 (1.02–1.29, p = 0.019)	1.17 (1.04–1.32, p = 0.0079)	
**Associations for social media time—weekday (hrs)**				
*Mental wellbeing*	1159	−0.65 (−0.84 to −0.47, p < 0.0001)	−0.50 (−0.69 to −0.32, p < 0.0001)	−0.15 (−0.21 to −0.09)
Anxiety	1159	0.49 (0.37–0.60, p < 0.0001)	0.39 (0.28–0.50, p < 0.0001)	0.19 (0.14–0.25)
Depression	1153	0.63 (0.51–0.76, p < 0.0001)	0.54 (0.41–0.66, p < 0.0001)	0.23 (0.18–0.29)
Problematic social media use	1152	3.10 (2.68–3.52, p < 0.0001)	2.84 (2.41–3.25, p < 0.0001)	0.36 (0.31–0.41)
*Sleep duration (minutes)*	728	−2.26 (−3.55 to −0.95, p = 0.00064)	−2.99 (−4.31 to −1.66, p < 0.0001)	−0.17 (−0.25 to −0.10)
*Sleep efficiency*	728	−0.17 (−0.31 to −0.02, p = 0.025)	−0.18 (−0.33 to −0.03, p = 0.018)	−0.10 (−0.17 to −0.01)
*Moderate-to-Vigorous PA*	751	−0.58 (−1.00 to −0.16, p = 0.0073)	−0.31 (−0.74 to 0.11, p = 0.17)	−0.05 (−0.13 to 0.02)
*Average daily acceleration*	751	−0.41 (−0.68 to −0.15, p = 0.0025)	−0.21 (−0.49 to 0.05, p = 0.12)	−0.06 (−0.13 to 0.01)
Disruptiveness	1045	0.10 (0.05–0.15, p < 0.0001)	0.11 (0.07–0.16, p < 0.0001)	0.13 (0.08–0.19)
Attainment English = below target^a^	1054	1.07 (1.02–1.13, p = 0.0082)	1.08 (1.03–1.14, p = 0.0034)	
Attainment Maths = below target^a^	1061	1.08 (1.03–1.14, p = 0.0026)	1.07 (1.02–1.13, p = 0.0094)	
**Associations for social media time—weekend day (hrs)**				
*Mental wellbeing*	1146	−0.58 (−0.72 to −0.44, p < 0.0001)	−0.44 (−0.58 to −0.30, p < 0.0001)	−0.18 (−0.24 to −0.12)
Anxiety	1146	0.40 (0.32–0.48, p < 0.0001)	0.31 (0.23–0.40, p < 0.0001)	0.21 (0.16–0.27)
Depression	1140	0.54 (0.45–0.63, p < 0.0001)	0.46 (0.36–0.55, p < 0.0001)	0.27 (0.21–0.33)
Problematic social media use	1140	2.16 (1.85–2.48, p < 0.0001)	1.90 (1.58–2.21, p < 0.0001)	0.33 (0.27–0.38)
*Sleep duration (minutes)*	720	−1.48 (−2.40 to −0.54, p = 0.0017)	−2.03 (−2.96 to −1.10, p < 0.0001)	−0.16 (−0.23 to −0.09)
*Sleep efficiency*	720	−0.11 (−0.21 to −0.01, p = 0.033)	−0.13 (−0.23 to −0.02, p = 0.014)	−0.09 (−0.17 to −0.02)
*Moderate-to-Vigorous PA*	744	−0.53 (−0.82 to −0.23, p = 0.00046)	−0.32 (−0.63 to −0.04, p = 0.034)	−0.08 (−0.15 to −0.01)
*Average daily acceleration*	744	−0.35 (−0.54 to −0.17, p = 0.00018)	−0.22 (−0.41 to −0.03, p = 0.024)	−0.08 (−0.15 to −0.01)
Disruptiveness	1033	0.06 (0.02–0.09, p = 0.0012)	0.07 (0.04–0.11, p = 0.00014)	0.11 (0.06–0.17)
Attainment English = below target^a^	1041	1.04 (1.00–1.08, p = 0.055)	1.05 (1.01–1.09, p = 0.17)	
Attainment Maths = below target^a^	1048	1.05 (1.01–1.09, p = 0.0096)	1.04 (1.00–1.09, p = 0.044)	
**Associations for social media time—week (hrs)**				
*Mental wellbeing*	1133	−0.10 (−0.13 to −0.08, p < 0.0001)	−0.08 (−0.11 to −0.05, p < 0.0001)	−0.17 (−0.23 to −0.11)
Anxiety	1133	0.08 (0.06–0.09, p < 0.0001)	0.06 (0.04–0.08, p < 0.0001)	0.22 (0.16–0.27)
Depression	1127	0.10 (0.08–0.12, p < 0.0001)	0.08 (0.07–0.10, p < 0.0001)	0.26 (0.21–0.32)
Problematic social media use	1127	0.46 (0.40–0.51, p < 0.0001)	0.41 (0.35–0.47, p < 0.0001)	0.38 (0.32–0.43)
*Sleep duration (minutes)*	713	−0.34 (−0.52 to −0.16, p = 0.00019)	−0.46 (−0.64 to −0.27, p < 0.0001)	−0.19 (−0.27 to −0.11)
*Sleep efficiency*	713	−0.02 (−0.04 to −0.00, p = 0.023)	−0.03 (−0.05 to −0.01, p = 0.010)	−0.10 (−0.18 to −0.02)
*Moderate-to-Vigorous PA*	736	−0.10 (−0.16 to −0.04, p = 0.00095)	−0.06 (−0.12 to 0.00, p = 0.063)	−0.07 (−0.14 to 0.00)
*Average daily acceleration*	736	−0.07 (−0.10 to −0.03, p = 0.00028)	−0.04 (−0.08 to 0.00, p = 0.043)	−0.07 (−0.15 to 0.00)
Disruptiveness	1022	0.01 (0.01–0.02, p < 0.0001)	0.02 (0.01–0.02, p < 0.001)	0.14 (0.08–0.20)
Attainment English = below target^a^	1030	1.01 (1.00–1.02, p = 0.019)	1.01 (1.00–1.02, p = 0.0052)	
Attainment Maths = below target^a^	1037	1.01 (1.00–1.02, p = 0.0034)	1.01 (1.00–1.02, p = 0.017)	

**Notes**: Wellbeing reported as WEMWBS score. Anxiety reported as GAD-7 score. Depression reported as PAQ-9 score. Problematic Social Media Use reported as PSMU score. Sleep Duration (hrs). Sleep Efficiency (%). Physical Activity (PA). Moderate-to-Vigorous PA (mins). Average Daily Acceleration (mg). Classroom Disruptiveness reported as PBQ score.

a Mixed effects logistic regression models; odd ratios reported for attainment below target vs attainment on or above target. The exposure difference of coefficients is 1 h. Italics represent positive outcomes, regular text represents adverse outcomes. School ID was included as a random effect in all models. N represents the N of the adjusted models. Year group was included as a random effect in all models except for the unadjusted Attainment models. Fixed effects variables included in the adjusted models at the individual-level were: sex, and ethnicity, and at the school-level were: %Pupils with English as an Additional Language (EAL), %Pupils with Special Educational Needs (SEN), %Pupils eligible for (FSM), school size, school Income Deprivation Affecting Children Index (IDACI), school religious affiliation, school admissions policy, school co-education status, and month of measurement.

Tables 4 and 5 show increased smartphone and social media time on weekend days and across the week was associated with decreased average daily acceleration and MVPA. A similar relationship was present for smartphone time, but not social media time and physical activity outcomes, on a weekday. There were no significant associations between smartphone and social media time in school and average daily acceleration or MVPA. Increased smartphone and social media time was associated with reduced sleep efficiency and sleep duration across all measured time periods, except for smartphone and social media time in school and for smartphone time on a weekday which only had a significant relationship to sleep duration but not efficiency.

Increases in smartphone and social media time, were associated with increases in disruptive classroom behaviour across all measured time periods (Table 4, Table 5). Increased smartphone and social media time was also associated with poorer English and Maths attainment across nearly all measured time periods (Table 4, Table 5).

## Discussion

There was no significant difference in adolescent mental wellbeing between pupils attending schools that permitted phone use compared to pupils attending schools that restricted phone use. However, there were negative associations between increasing time spent on phones/social media and lower mental wellbeing. Similarly, we observed no significant differences in anxiety, depression, problematic social media use, sleep, physical activity, attainment, and disruptive behaviour when comparing adolescents exposed to restrictive or permissive school phone policies, but we did observe significant negative associations between these outcomes and increasing phone and social media time. This study therefore provides further evidence of the adverse consequences from increased smartphone and social media use, and that lowering phone and social media use is important.

There was some variability on the time adolescents spent on phones and social media during the school day within restrictive and permissive school policy groups, and this further supports our previously reported findings that pupils' and teachers’ understanding and adherence to school phone policies varies.^26^ Overall, adolescents attending schools with restrictive phone policies spent less time on their phones and social media during their time in school (e.g., 9 am–3 pm). However, this reduced use in schools with restrictive phone policies did not manifest in differences in the overall time spent on phones and social media or differences in mental health and wellbeing and other associated outcomes, even for adolescents attending restrictive schools where phones were inaccessible to them during the school day (e.g., phones stored in a locker, a pouch, at school reception or left at home). This finding could be partially explained by the lack of difference between permissive and restrictive schools in overall phone/media use duration during a full day or week. Similar to the SCAMP study,^28^ data shows that in-school phone and social media use is only a small contributor to overall use on school days and at weekends, and even when weekend use is controlled for, there are no observed differences in outcomes. Furthermore, in-school and out of school phone use are positively correlated, which suggests that in-school and out of school phone/media use behaviours are comparable.

Overall, our findings suggest that restrictive school policies in their current form do not significantly influence phone and social media use or result in better outcomes for adolescents across a range of mental, physical, and cognitive domains. These findings therefore do not provide evidence to support the use of school policies that prohibit phone use during the school day in their current form, and indicate that these policies require further development and linking with the wider context to enable a more comprehensive approach to reducing overall phone and social media use in adolescents, addressing both in-school and out of school use. Additionally, future research may explore other in-school behaviours that may benefit from a restrictive school phone policy, such as face-to-face interactions or bullying,^19^ and/or whether different policy types within restrictive categories or length of policy implementation influence outcomes.

We are not aware of other peer-review published studies that have examined the relationship between school phone policies and mental health and wellbeing, and related behavioural outcomes. There are several longitudinal, ecological, and cross-sectional studies that report non-linear U-shaped relationships between screen-based activities and mental health outcomes.^15^^,^^16^ We, along with other recent studies,^21^^,^^22^ identified a linear relationship, which could be explained by the median phone time (see Supplementary File) in our adolescent sample being much greater (4–6 h per day) than the previously reported cut offs for experiencing benefits (<2 h per day) and risks (>2 h per day) to mental health.^14^, ^15^, ^16^, ^17^, ^18^, ^19^, ^20^ It is worth noting this extreme increase in user duration among adolescents within the last decade, and particularly since 2016 when the daily duration of UK adolescents phone and social media use was estimated to be 2 h.^10^ Our study is also one of the first to look specifically at smartphone and social media time, rather than generic screen time. However, time is only one measure of phone/social media use. Similar to other studies,^10^^,^^21^^,^^22^ future research should measure other features of phone use (e.g., gaming and calling) and specific uses across multiple devices (e.g., social media use on phones, tablets, laptops) to provide further data about the interactions between phones/social media and wellbeing, and to explore variations in effects in populations where social media is not the dominant type of phone use. Furthermore, it is important to consider other measures of problematic phone and social media use to comprehensively assess phone/media use behaviours, such as those measures more closely linked to the DSM-V criteria of behavioural addiction.^35^ There is also scope to consider other methods of data collection to enhance the accuracy of reporting for the outcome measures, such as the use of sleep polysomnography instead of accelerometer or self-reported measures of sleep.

This study used a cross-sectional observational approach to explore the potential effects of school phone policies in a large nationally representative cohort, using regression analysis to control for possible confounding factors. The recruited schools differed in several ways from those in the sampling frame (e.g., academy status, selective, sixth form, faith), and given the school level response rates, this may have been a source of selection bias impacting on the generalizability of the findings. There were also some differences in the permissive and restrictive sample of schools (e.g., a greater proportion of selective, single sex and secular schools in the permissive school category), and this reflected the differences in the permissive and restrictive schools in the sampling frame. School characteristics were controlled for in the analyses. Overall, therefore, despite some sample limitations, the results from this study are likely to be applicable across the UK. A limitation is that the study design is cross sectional which makes it difficult to draw conclusions about causality and reverse causality cannot be ruled out. Furthermore, there is a risk of selection bias and unmeasured confounding in this study. For example, other school characteristics may impact on the outcomes, possibly having a confounding effect.

The self-reported data on phone and social media use duration collected in this study may have introduced a bias; pupils in schools with restrictive phone policies may have been more likely to under-report their in-school phone use, compared with those in schools with permissive policies. If this were the case, the difference in in-school use between the two groups that we report would be an over-estimate. Data were collected from adolescent phones on screen time and social media use; however, we were unable to include these measures in our analyses, due to concerns related to the accuracy of adolescents self-reporting phone and social media data from apps, as well as a high proportion of implausible data. Future studies should explore ways to access for research purposes the data that are available on adolescents’ phones. Our questionnaire did not ask adolescents if they did not have a smartphone, and future studies should explore how outcomes in non-users compare. Gender identity groups that differ from sex registered at birth should be explored further, as in this study the group was too small to incorporate as an adjustment variable to say anything in confidence.

In conclusion, school phone policies that restrict the daytime use of phones lower the time adolescents spend on phones/social during their time in school but are not associated with an overall reduction in the time adolescents spend on phones and social media. In addition, there is no evidence to support that restrictive school phone policies, in their current forms, have a beneficial effect on adolescents' mental health and wellbeing or related outcomes, indicating that the intentions of these policies to improve adolescent health, wellbeing, and educational engagement are not realised. Our data suggest that interventions to reduce phone/social media time to positively influence adolescent mental wellbeing are plausible, but that both in-school and outside of school use should be considered in tandem. Preventative efforts should also consider how other behaviours that influence wellbeing are influenced by increased phone/media use, such as sleep, physical activity, attainment, classroom behaviour, and problematic use. In the design of new guidelines and interventions, phone and media use could be approached as part of a ‘compositional whole’, whereby phone/media time guidance focuses on obtaining the ‘right balance’ between time spent on devices and other daily lifestyle behaviours. This approach does not necessarily preclude restrictive school mobile phone policies, but these policies would be linked with a wider holistic approach to adolescent mobile phone and social media use. This is comparable to the 24-h model taken in the study of movement behaviours, and in the design of physical activity guidelines.

## Contributors

VG and MP conceptualised the study and were responsible for the overall study design, funding acquisition, study leadership and writing of the manuscript. AR led recruitment and data collection, supervised the research team, prepared the data for analysis, and contributed to the writing of the original manuscript drafts. With input from AS, BM conducted the statistical analysis and prepared figures, tables and supplementary material. SF and JQ led the analysis and reporting of accelerometer data. RT supported school-based data collection and the preparation of data for analysis. PA, HA, KJ, MM, PP, and MW contributed to the overall study design and reviewing and editing of the manuscript. All authors had full access to all the data in the study and had final responsibility for the decision to submit for publication. VG, AR and BM have accessed and verified the data.

## Data sharing statement

The SMART Schools Study investigator team will consider reasonable requests for the sharing of fully anonymised study data, and data dictionaries. The data will be made available following the grant period, and after the acceptance of the report to the funder (estimated 2026). Requests should be submitted to the corresponding author (v.a.goodyear@bham.ac.uk), and a signed access agreement will be made.

## Declaration of interests

VG: National Institute for Health and Care Research (NIHR) Public Health Research Programme funding was received for the reported work (NIHR131396). All funding is paid to The University of Birmingham. MP: NIHR Public Health Research Programme funding was received for the reported work (NIHR131396). All funding is paid to The University of Birmingham. AS: holds an NIHR grant and is supported by the NIHR Birmingham Biomedical Research Centre. Her funding is paid to the University of Birmingham. HA: HA holds a Wellcome Trust Investigator Award in Humanities and Social Sciences to investigate wellbeing investment in schools and workplaces. The award is paid to University of Birmingham. All other authors declare no competing interests.

## Appendix A Supplementary data

Supplementary Figures and Tables

Published Protocol SMART Schools Study
