# Supplementary material for: School phone policies and their association with mental wellbeing, phone use, and social media use (SMART Schools): a cross-sectional observational study
Source: Lancet Reg Health Eur. 2025 Feb 4;51:101211. doi: 10.1016/j.lanepe.2025.101211 (PMC11984610; doi:10.1016/j.lanepe.2025.101211)
Supplement: Supplementary Figures and Tables [file mmc1.docx]

**Supplementary Appendix: School phone policies, phone and social media use, and the association with mental health and wellbeing, sleep, physical activity, and educational outcomes: the SMART Schools cross sectional observational study.**

Contents

[Supplementary Figure 1: Logic Model and theory of change for the influence of school phone policies that restrict daytime use of smartphones on mental wellbeing and health outcomes in adolescents 2](#_Toc185494686)

[Supplementary Table 1: Outcome measures and handling of missing data 3](#_Toc185494687)

[Adjustment Variables 7](#_Toc185494688)

[Data Cleaning Notes 8](#_Toc185494689)

[Accelerometer Data Processing 10](#_Toc185494690)

[Supplementary Figure 2: Correlations between self-reported phone data and self-reported smartphone/social media use. 11](#_Toc185494691)

[Supplementary Figure 3: Correlations between Mental Health and Wellbeing Outcomes and Smartphone and Social Media Time 12](#_Toc185494692)

[Supplementary Figure 4: Correlations between Sleep and Physical Activity (PA) Outcomes and Smartphone and Social Media Time 13](#_Toc185494693)

[Supplementary Table 2: Characteristics of the Sampling Frame and Recruited Sample 15](#_Toc185494694)

[Supplementary Table 3: Time current smartphone policies have been in place (Randhawa et al., 2024) 16](#_Toc185494695)

[Supplementary Table 4: Exploration of Interaction Effects Between Phone Policy and school Income Deprivation Affecting Children Index (IDACI), pupil Sex, pupil ethnicity, and pupil Year Group 17](#_Toc185494696)

[Supplementary Table 5 Adjusted Mean Differences in Mental Wellbeing by School Phone Policy (restrictive vs permissive phone policy group): Sensitivity Analysis 18](#_Toc185494697)

[Supplementary Figure 5: The Distribution of Smartphone and Social Media Use Duration (hrs) in School in Permissive and Restrictive Schools 19](#_Toc185494698)

[Supplementary Table 6: Adjusted Mean Differences in Outcomes by School Phone Policy (restrictive vs permissive phone policy group [reference] compared with restrictive inaccessible school phone policies vs permissive phone policy group [reference]): Sensitivity Analysis 20](#_Toc185494699)

[Supplementary Table 7. Participant-level Outcome Data Summarised by School Group 21](#_Toc185494700)

[Supplementary Table 8 Adjusted Mean Differences in Outcomes by School Phone Policy (restrictive vs permissive phone policy group) controlling for weekend day smartphone time: Sensitivity Analysis 23](#_Toc185494701)

[Supplementary Figure 5: Pearson Correlation between Self-Report Smartphone Time In-School, across the School Day and for a Weekend Day 24](#_Toc185494702)

[Supplementary Figure 6: Pearson Correlation between Self-Report Social Media Time In-School, across the School Day and for a Weekend Day 25](#_Toc185494703)

[Supplementary Figure 7: Proportion of pupils reporting higher social media time than phone screen time from self-reported phone data between permissive and restrictive schools 26](#_Toc185494704)

[Summary of Ethical Procedures for Opt Out Consent 27](#_Toc185494705)

[Pupil Survey 28](#_Toc185494706)

[STROBE Checklist 46](#_Toc185494707)

# Supplementary Figure 1: Logic Model and theory of change for the influence of school phone policies that restrict daytime use of smartphones on mental wellbeing and health outcomes in adolescents

**Context**

Smartphone and Social Media use is prevalent during adolescence.

Problematic social media use (prevalence 3-14%)

Increasing time on smartphones and social media may lead to decreasing levels of mental wellbeing and higher anxiety and depression.

Adolescents with poor mental health and wellbeing, sleep and physical activity are likely to spend more time on their phones and social media.

**Restrict Smartphone Use**

*(Assessed through document/website analysis and surveys)*

School policies where smartphone use is not permitted during the school day.

**Altered Phone/Social Media Use/Motives (intermediary outcomes)**

*(assessed through phone/app data and survey)*

Lower daily phone screen time

Lower social media time

Externally driven motives for use (e.g. social interaction)

**Behavioural Outcomes**

*(Assessed through accelerometers and surveys)*

Increased time in physical activity overall and moderate-to-vigorous physical activity

Increased sleep duration and efficiency

Less disruptive classroom behaviour

Higher attainment scores

Lower prevalence of problematic use

**Mental Health/Wellbeing Outcomes**

*(Assessed through surveys)*

Greater Mental Wellbeing

Lower Anxiety

Lower Depression

**Direct effect**

Reduced time available for phone/social media access

**Indirect effect**

Altered social norms and expectations.

# Supplementary Table 1: Outcome measures and handling of missing data

| **Outcome** | **Outcome measure** | **Timepoint(s) of evaluation** | **Scoring/Unit of measurement** | **Missing data** |
| --- | --- | --- | --- | --- |
| **Primary Outcome** | | | | |
| Mental well-being  (Pupil survey) | Warwick-Edinburgh Mental Well-Being Scale (WEMWBS)  Waite M, Atkinson C. A review of mental health and wellbeing measures for universal secondary school screening. Emotional and Behavioural Difficulties. 2021;26(2):206-22. | Measured over the past 2 weeks at two time points, 4–8 weeks apart | A total score is calculated at each time point, ranging from 14 to 70, with higher scores indicating higher well-being.  An overall score is calculated for each participant by averaging their total scores at both time points.  Scores can be further categorised as follows:  Low well-being 14-42  Medium wellbeing 43-59  High wellbeing 60-70 | If three or less responses are missing, then impute these by using the average of the other non-missing responses. If four or more responses are missing do not impute.  If one of these scores across the timepoints is missing, then use the one available total score as the overall score. |
| **Secondary Outcomes (health and education)** | | | | |
| Anxiety symptoms  (Pupil survey) | Generalised Anxiety Disorder Assessment (GAD-7)  Löwe B, Decker O, Müller S, Brähler E, Schellberg D, Herzog W, et al. Validation and standardization of the Generalized Anxiety Disorder Screener (GAD-7) in the general population. Med Care. 2008;46(3):266-74. | One time point measured over the past 2 weeks | A total score is calculated ranging from 0 to 21, with higher scores indicating higher anxiety.  Scores can be further categorised as follows:  Minimal anxiety 0-4  Mild anxiety 5-9  Moderate anxiety 10-14  Sever anxiety 15-21 | If one response is missing, then impute by using the average of the non-missing responses. If two or more are missing, then do not impute. |
| Depressive symptoms  (Pupil survey) | Patient Health Questionnaire (PHQ-9)  Kroenke K, Spitzer RL, Williams JB. The PHQ-9: validity of a brief depression severity measure. J Gen Intern Med. 2001;16(9):606-13. | One time point measured over the past 2 weeks | A total score is calculated ranging from 0 to 27, with higher scores indicating higher depression.  Scores can be further categorised as follows:  Minimal depression 0-4  Mild depression 5-9  Moderate depression 10-14  Moderately severe depression 15-19  Severe depression 20-27 | If one response is missing then impute by using the average of the non-missing responses. If two or more are missing, then do not impute. |
| Problematic use  (Pupil survey) | Problematic Social Media Use Scale (PSMU)  Marino C, Vieno A, Moss AC, Caselli G, Nikčević AV, and Spada MM. Personality, motives and metacognitions as predictors of problematic Facebook Use in university students. Personality and Individual Differences. 2016;101:70-77. | One time point based on usual use | A total score is calculated ranging from 15 to 120, with higher scores indicating more problematic use. This is calculated by adding up the scores from the five subscales. | If three or less missing responses overall then impute those using the average of the other responses in that subscale – but a maximum of one missing item per subscale. |
| Sleep quality  (Accelerometers) | Sleep duration | Over 7 days | Unit: hours | Not imputed  Missing data is very typical for accelerometery, and imputation (within-person – i.e. if a person is missing a few days of data), is not recommended/done with this type of data. The argument is that reliability studies show intra-class correlation coefficients of >0.8 where accelerometers have been worn ≥4 days, which means the data reliably captures a person’s typical physical activity patterns. Imputing data across participants – i.e. where someone has no accelerometer data, and we impute based on the sample average, introduces a level of bias when exploring associations with outcomes (e.g., mental wellbeing). Therefore a minimum of 4 days of wear time was selected and is representative of one’s physical activity pattern. Supporting literature for these points are provided here:  Rowlands, A. V., Pilgrim, E. L., & Eston, R. G. (2009). Seasonal changes in children’s physical activity: An examination of group changes, intra-individual variability and consistency in activity pattern across season. Annals of Human Biology, 36(4), 363–378. https://doi.org/10.1080/03014460902824220  Rowlands, A. V., Plekhanova, T., Yates, T., Mirkes, E. M., Davies, M., Khunti, K., & Edwardson, C. L. (2019). Providing a Basis for Harmonization of Accelerometer-Assessed Physical Activity Outcomes Across Epidemiological Datasets. Journal for the Measurement of Physical Behaviour, 2(3), 131-142. Retrieved Nov 28, 2024, from https://doi.org/10.1123/jmpb.2018-0073  da Silva SG, Evenson KR, da Silva ICM, et al. Correlates of accelerometer-assessed physical activity in pregnancy—The 2015 Pelotas (Brazil) Birth Cohort Study. Scand J Med Sci Sports. 2018; 28: 1934–1945. https://doi.org/10.1111/sms.13083  In our sample daily wear time was also high. On average watches were worn for 98.24% of the time in the days included in the analysis. This approximately equates to 30 minutes each day on average of non-wear time, so very minimal. This means that we didn’t have much missing data within a day that would require imputation.  Our approach to the analysis of accelerometer data is therefore in keeping with typical approaches to analysing accelerometer data. |
|  | Sleep efficiency | Over 7 days | Unit: percentage - percent of time asleep versus percent of time in bed |  |
| Physical Activity  (Accelerometers) | Moderate-to-vigorous physical activity (MVPA) | Over 7 days | Unit: minutes - the total time (mins) spent at above either moderate or vigorous thresholds, averaged across all valid days |  |
|  | Average daily acceleration (overall physical activity) | Over 7 days | Unit: mg - an indicator of total physical activity/movement, averaged across all valid days |  |
| Attainment  (Teacher Survey) | Pupils are rated as below, on, or above target in Maths and English | One time point— most recent assessment | Responses were categorised into ‘on or above target’ vs ‘below target’ to create a binary variable | N/A – not imputed |
| Disruptive classroom behaviour  (Teacher Survey) | Pupil Behaviour Questionnaire  Allwood M, Allen K, Price A, Hayes R, Edwards V, Ball S, et al. The reliability and validity of the pupil behaviour questionnaire: a child classroom behaviour assessment tool. Emotional and Behavioural Difficulties. 2018;23(4):361-71 | One time point—current assessment | A total score is calculated ranging from 0 to 12, with higher scores indicating more disruptive behaviour. | If one response is missing, then impute by using the average of the non-missing responses. If two or more are missing, then do not impute. |
| **Secondary outcomes (smartphone and social media use)** | | | | |
| Motives for social media use  (Pupil Survey) | Social Media Use Motives Questionnaire  Marino C, Vieno A, Moss AC, Caselli G, Nikčević AV, and Spada MM. Personality, motives and metacognitions as predictors of problematic Facebook Use in university students. Personality and Individual Differences. 2016;101:70-77 | One time point measured over past 12 months | There are 4 subscales within the questionnaire representing different motives for social media use: Coping, Conformity, Enhancement, and Social. A total is calculated for each subscale ranging from 4 to 20, with a higher score indicating higher motivation for this subscale. | For each subscale total, if one response is missing within that subscale then impute using the average of the other responses in that subscale. If two or more responses are missing, do not impute. |
| Self-reported smartphone use duration  (Pupil survey) | Smartphone Time – In School (within school hours) | One time point, based on usual use | Units: hours | If either the self-reported smartphone use or the self-reported social media use during the school day was greater than the time at school both variables were set to NA. Additionally, if self-reported social media time was greater than self-reported screen time then the screen time was set to match the social media time. |
|  | Smartphone Time – Weekday (over 24 hours on a weekday) | One time point, based on usual use | Units: hours | If either the self-reported smartphone use or self-reported social media use is greater than 22 hours then that was assumed to be an error and both of the measures were put as NA. Additionally, is self-reported social media time was greater than self-reported smart phone use was the number used for self-reported smartphone use. Additionally, if self-reported social media time was greater than self-reported screen time then the screen time was set to match the social media time. |
|  | Smartphone Time – Weekend Day (over 24 hours on a weekend day) | One time point, based on usual use | Units: hours |  |
|  | Smartphone Time – Week (over a full (7-day) week) | One time point, based on usual use | Units: hours – this was calculated as a total of 5 x school day use and 2 x weekend day use. | If either the school day or weekend day measure for the self-reported smartphone use was NA this was set to NA. |
| Self-reported social media use duration  (Pupil survey) | Social Media Time – In School (within school hours) | One time point, based on usual use | Units: hours | If either the self-reported smartphone use or the self-reported social media use during the school day was greater than the time at school both variables were set to NA. |
|  | Social Media Time – Weekday (over 24 hours on a weekday) | One time point, based on usual use | Units: hours | If either the self-reported smartphone use or self-reported social media use is greater than 22 hours then that was assumed to be an error and both of the measures were put as NA. |
|  | Social Media Time – Weekend Day (over 24 hours on a weekend day) | One time point, based on usual use | Units: hours |  |
|  | Social Media Time – Week (over a full (7-day) week) | One time point, based on usual use | Units: hours – this was calculated as a total of 5 x school day use plus 2 x weekend day use. | If either the school day or weekend day measure for the self-reported social media use was NA this was set to NA. |
| Self-reported phone data  (Pupil survey: Reported from data captured from Screentime app on iOS or Digital Wellbeing app on Android | Smartphone Time – Weekday (over 24 hours on a weekday) | One time point, based on previous full school day | Units: hours | If either the screen time or social media time was greater than 22 hours then that was assumed to be an error and both of the measures were put as NA. |
|  | Smartphone Time – Weekend Day (over 24 hours on a weekend day) | One time point, based on one full day of previous weekend | Units: hours |  |
|  | Smartphone Time – Week (over a full (7-day) week) | One time point, based on use in previous week | Units: hours – this was calculated as a total of 5 x school day use and 2 x weekend day use. | If either of the school day or weekend phone reported screentime measures were NA this was set to be NA. |
| Self-reported social media data  (Pupil survey: Reported from data captured from Screentime app on iOS or Digital Wellbeing app on Android | Social Media Time – Weekday (over 24 hours on a weekday) | One time point, based on previous full school day | Units: hours – calculated as the sum of the reported time on each individual social media app | If either the screen time or social media time was greater than 22 hours then that was assumed to be an error and both of the measures were put as NA. Additionally, if social media time was greater than the corresponding screen time measure than social media time was set to NA. |
|  | Social Media Time – Weekend Day (over 24 hours on a weekend day) | One time point, based on one full day of previous weekend | Units: hours – calculated as the sum of the reported time on each individual social media app |  |
|  | Social Media Time – Week (over a full (7-day) week) | One time point, based on use in previous week | Units: hours – this was calculated as a total of 5 x school day use plus 2 x weekend day use. | If either of the school day or weekend social media measures were NA this was set to be NA. |

# Adjustment Variables

School-level and pupil-level covariates were included as fixed and random effects variables in the models when comparing outcomes between the two school phone policies (research question 1a, 1b) and the associations between smartphone/social media time and outcomes (research question 2):

- ***School Level:*** School ID; School Size; Income Deprivation Affecting Children Index (IDACI); Religious Affiliation (religious vs secular); Admissions Policy (selective vs non-selective); Coeducation status (mixed vs single sex); Percentage of pupils with Special Educational Needs (SEN); Percentage of pupils with English as an Additional Language (EAL); Percentage of pupils eligible for Free School Meals (FSM); Month of measurement.
- ***Pupil Level:*** Year group (year 8, age 12-13, or year 10, age 14-15); Sex – self-reported in pupil survey (pupil participants were asked *“what is your sex?”* with the response options *“female”, “male”,* or *“I would rather not say”*); Ethnicity (self-reported in pupil survey using 2021 England and Wales Census classifications which were combined into the following categories: White; Mixed/Multiple; Asian/Asian British; Black/African/Caribbean/Black British; Other/Prefer not to say/Missing).

# Data Cleaning Notes

**Warwick-Edinburgh Mental Wellbeing Scale (WEMWBS):**

Calculation for the Warwick-Edinburgh Mental Wellbeing Scale (WEMWBS) involved summing scores for 14 questions that ranged from 1 (none of the time) to 5 (all of the time) with higher scores indicating higher wellbeing. If there was a single question missing, then that question was inputted using the average of other questions which affected 111 participants. If there were more than 1 question missing, then no score was computed which affected 4 participants. Since two WEMWBS scores were taken the average of both were used if participants had both scores (n = 952) or if they only had one score complete (n = 271) then that was used.

**Anxiety (GAD-7) and Depression (PHQ-9):**

Calculation for both the anxiety and depression scales involving adding up 7 questions for anxiety (GAD-7) and 9 questions for depression (PHQ-9) with options ranging from 0 (not at all) to 3 (nearly every day) with higher scores indicating higher anxiety or depression. If a participant missed one question within a scale (this happened to 18 participants for the anxiety measure and 34 for the depression measure) then that question was imputed using the average of the other questions within the scale and a total was calculated. If there were more than 1 question missing within a scale (happened to 13 for the anxiety scale and 20 for depression) then their scores were not included.

**Problematic Social Media Use Scale (PSMU):**

The Problematic Social Media Use scale (PSMU) is 15 questions across 5 subscales. The scores range from 1 (definitely disagree) to 8 (definitely agree) with higher scores indicating more problematic social media usage. An overall score is created by adding together the 5 subscales that have 3 questions each. If a subscale has only one missing question then that question is imputed using the other 2 questions with 35 participants having at least one question being imputed. If there are more than 4 subscales that needed to be imputed then those scores were not included. Overall, there were 28 scores that were missing.

**Social Media Use Motivation (SMUM):**

Motivation for social media use behaviours was measured using the SMUM which focuses on 4 motivations: coping, conformity, enhancement, and social. All 4 were computed by adding together 4 questions that ranged from 1 (never) to 5 (always) with higher scores indicating that field being a higher motivation for social media use. For each subscale, if one question was missing then that question was imputed using the remaining questions within the subscale. This affected 17 for the coping measure, 14 for conformity, 36 for enhancement, and 17 for the social subscale.

**Disruptive Scale:**

The disruptive score was calculated by asking a student’s teacher 6 questions about their behaviour with options ranging from 0 (never) to 2 (frequently). If a single question is missing, then the score for that question was imputed using the average of other questions which affected 12 participants.

**Self-Reported Phone Reported Smartphone Time and Social Media Usage:**

There was some basic cleaning done to the phone use times as there were some text options when looking at time. The text “30 minutes” was changed to 30 and the text “time was around 2 hours” was put at 2 hours. There was also a tyt6 text and this was changed to just 6. A 4o was changed to 40.

Phone reported Smartphone Time and social media use was collected by instructing participants to go to their phone’s digital well-being app or screen time ios app to access numbers from a single school day and a single weekend day. Due to typical input errors as well as some issues with how the digital well-being app may be counting social media time there were some issues with the data which resulted in some cleaning.

Firstly, when the time spent on phone or on social media was reported to be more than 22 hours then the numbers for that day (for both social media and Smartphone Time counts, even if only one of these was an implausible value) were excluded. For the school day measures, this resulted in removing data from 29 participants. For the weekend measures, this results in removing data from 31 participants.

Additionally, due to the way the app may have been miscounting social media time, 27·79 percent of participants had higher social media times than Smartphone Times for their phone reported school day data and 15·48 percent of participants had higher social media times than Smartphone Times for their phone reported weekend day data. When this happened, their social media time was excluded from analysis affected 341 participants school day social media data and 190 participants weekend day social media data.

**Self-Reported Smartphone Time and Social Media Usage:**

For self-reported phone use, we also exclude values which we viewed as implausible. For school day and weekend data this also involved excluding values where with the social media or the Smartphone Time was more than 22 hours which affected 3 participant’s school day data and 12 participant’s weekend day data. For self-reported in school phone use, we used the arrival at school and departure from school times given to calculate the total time spent at school and then if either the self-report Smartphone Time in school or the self-reported social media time in school was more than that value both data points were excluded which affected 47 people.

When social media time was greater than Smartphone Time, instead of excluding it like we did for the phone reported data, we set the Smartphone Time to equal the social media time as previous research has found that self-reported social media time tended to be more accurate than self-reported Smartphone Time. This affected 12·39% of participant’s self-reported school day Smartphone Time data, 9·21% of participant’s self-reported weekend Smartphone Time data, and 13·85% of participant’s in school Smartphone Time data.

**Demographic Variables**

For 120 missing IMDs for students the median IMD for school was used instead. For 3 people with missing age the median age by school and year group was used instead. This method was also used to replace the number for someone with the age of 93 as that was assumed to be an error.

# Accelerometer Data Processing

Extracted accelerometer files were processed and analysed with an open-source R package, GGIR (version 2·5-0, http://cran.r-project.org)^1^ and quality control cleaning occurred via STATA SE (version 18·0). Data was calculated for each valid day and averaged across all valid days for each pupil. Invalid days were classed as those which failed calibration or where no wear occurred. Nights were deemed invalid if sleep duration was less than 3 hours or longer than 16 hours. A minimum of 4 valid days were required for analysis.

^1^Rowlands AV, Yates T, Davies M, Khunti K, Edwardson CL. Raw accelerometer data analysis with GGIR R-package: Does accelerometer brand matter? Medicine and Science in Sports and Exercise. 2016; 48(10):1935–1941

# Supplementary Figure 2: Correlations between self-reported phone data and self-reported smartphone/social media use.


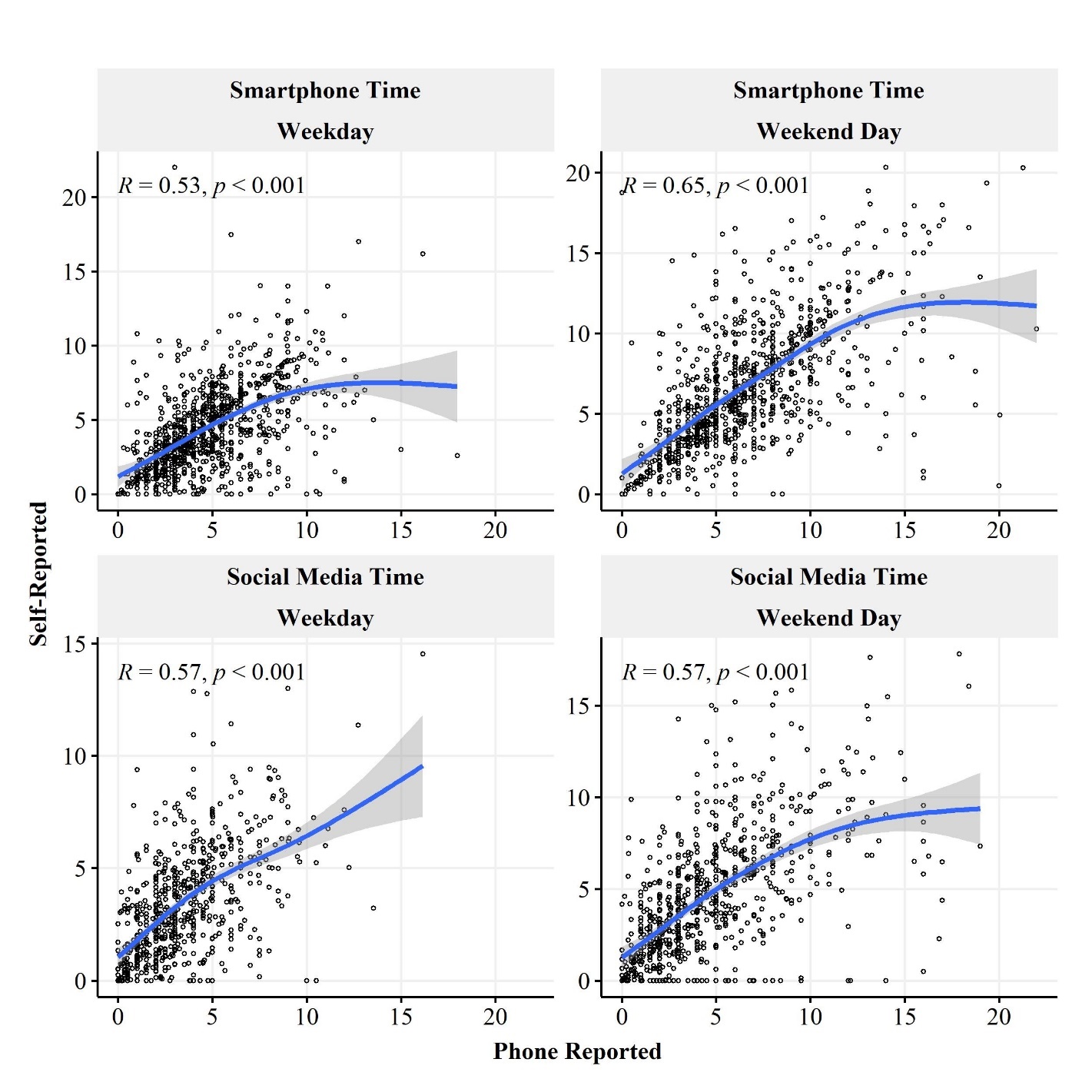


#
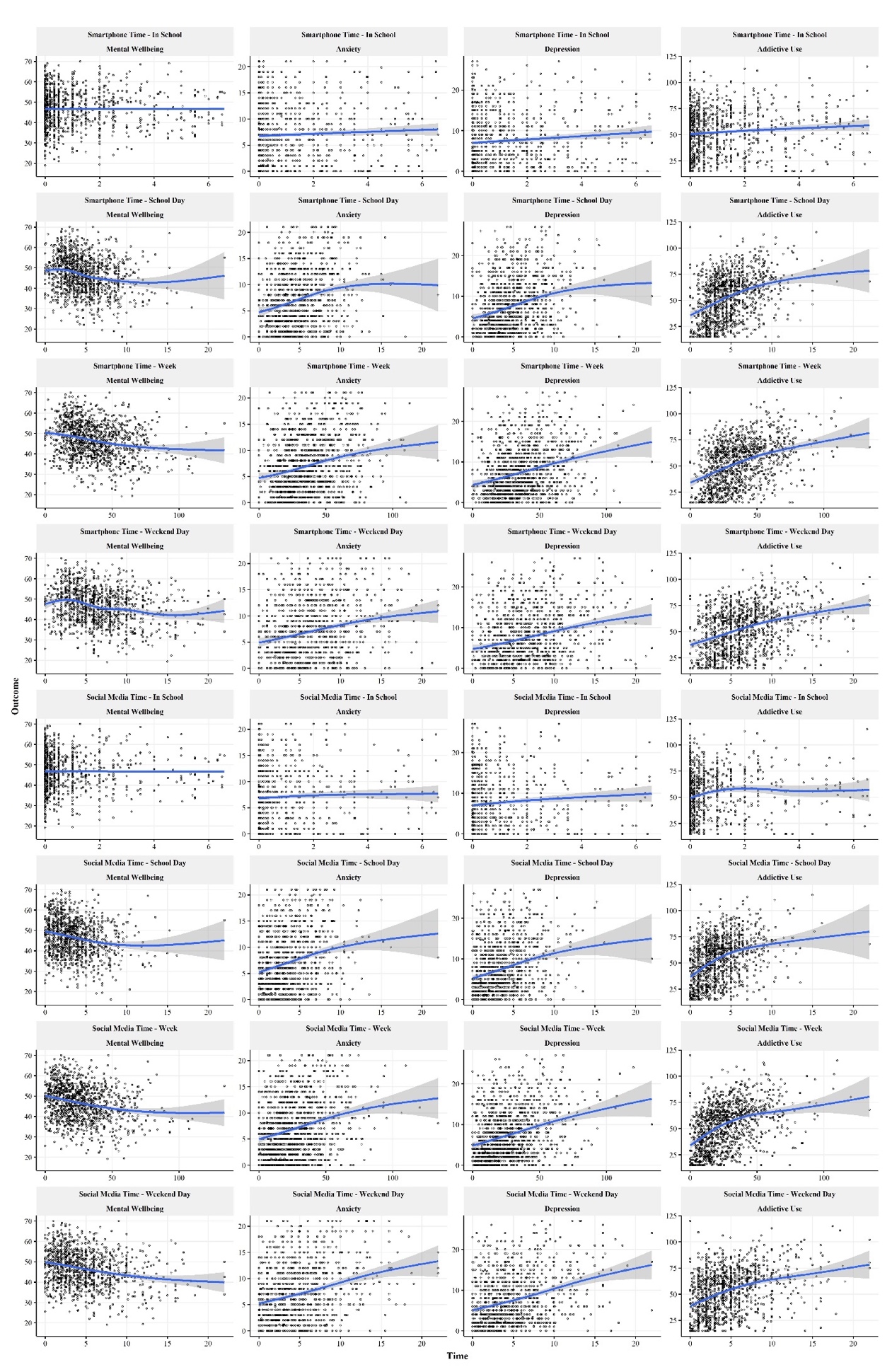
Supplementary Figure 3: Correlations between Mental Health and Wellbeing Outcomes and Smartphone and Social Media Time

#
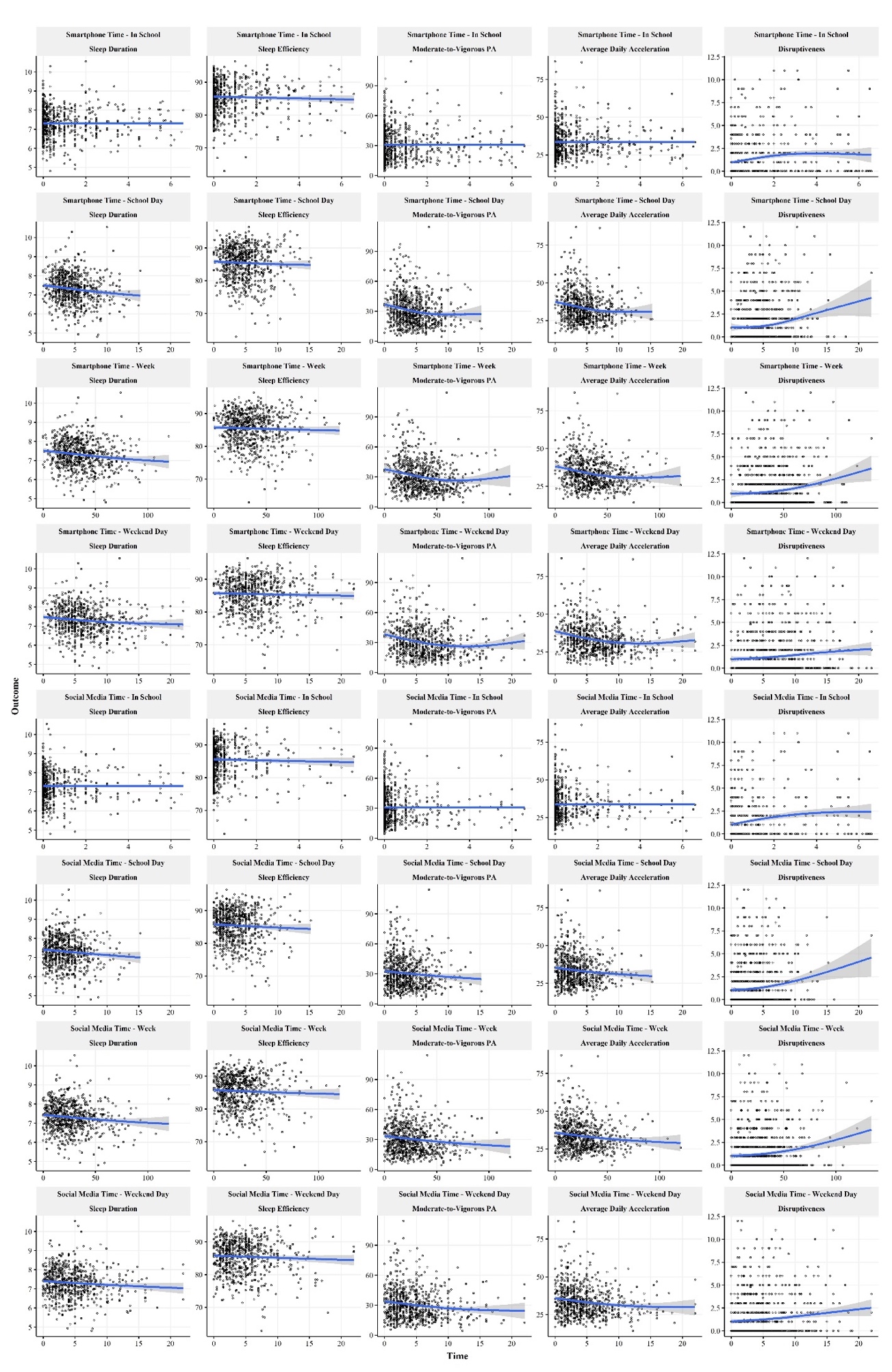
Supplementary Figure 4: Correlations between Sleep and Physical Activity (PA) Outcomes and Smartphone and Social Media Time


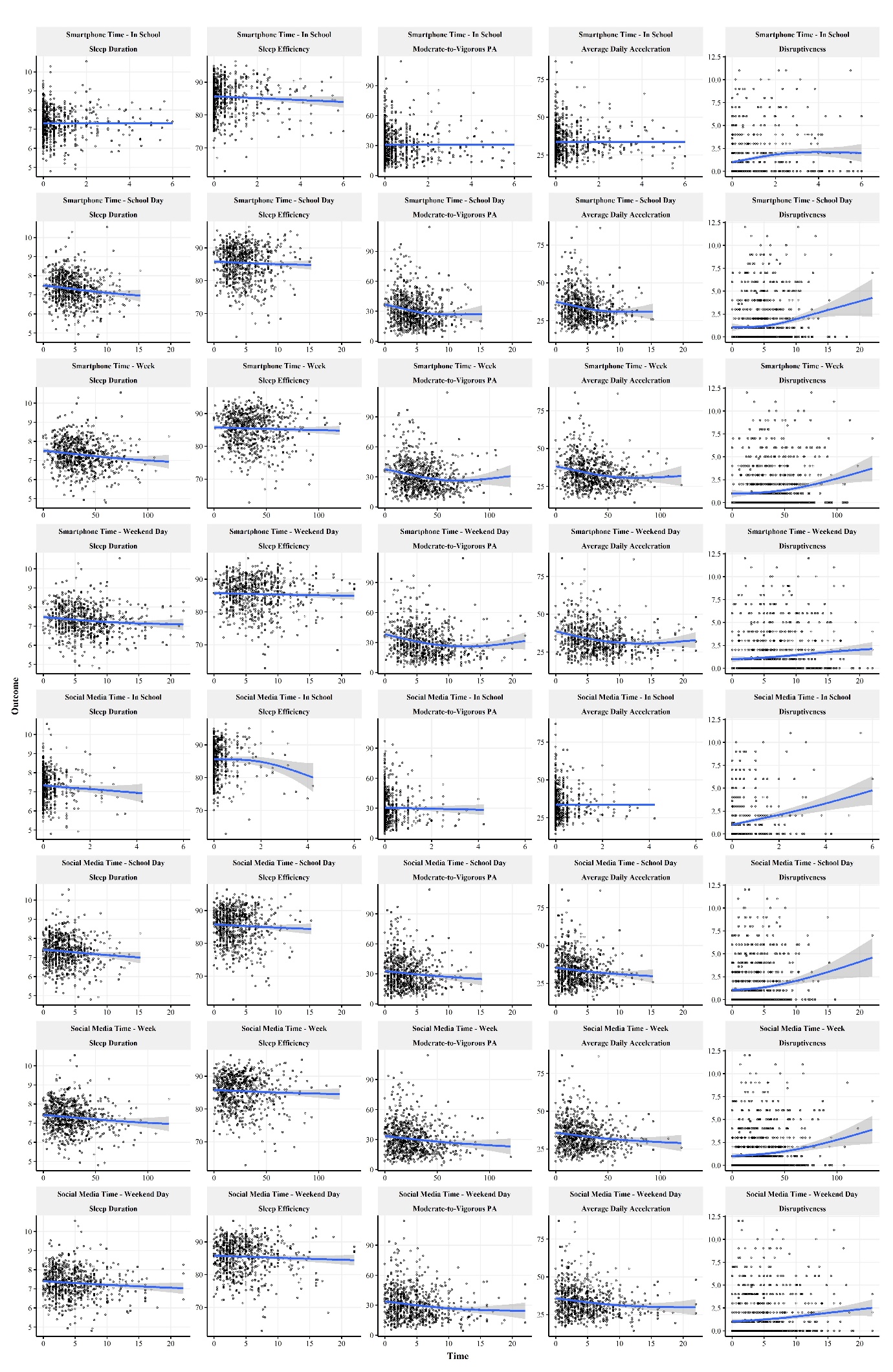


# Supplementary Table 2: Characteristics of the Sampling Frame and Recruited Sample

| **Characteristics** | **All Schools** | | **Schools with Restrictive Phone Policy** | | **Schools with Permissive Phone Policy** | |
| --- | --- | --- | --- | --- | --- | --- |
|  | **Sampling frame**  **(n =1341)** | **Recruited (n=30)** | **Sampling Frame**  **(n = 1245)** | **Recruited (n=20)** | **Sampling Frame**  **(n= 96)** | **Recruited (n=11)** |
| Academy Converter | 707 (52·72%) | 22 (73·33%) | 656 (52·69%) | 14 (70·00%) | 51 (53·13%) | 8 (80·00%) |
| Urban | 1153 (85·98%) | 25 (83·33%) | 1075 (86·34%) | 17 (85·00%) | 78 (81·25%) | 8 (80·00%) |
| Single Sex Schools | 130 (9·69%) | 3 (10·00%) | 117 (9·40%) | 1 (5·00%) | 13 (13·54%) | 2 (20·00%) |
| Pupils from Black or Minority Ethnic Groups % | 33·86% | 32·56% | 33·95% | 30·89% | 32·64% | 35·91% |
| Pupils with EAL* % | 16·92% | 15·87% | 16·92% | 15·67% | 16·92% | 16·29% |
| Pupils Eligible for FSM** % | 22·36% | 17·90% | 22·45% | 18·85% | 21·29% | 16·00% |
| Pupils Eligible for SEN*** Support % | 12·50% | 11·80% | 12·50% | 13·00% | 12·00% | 10·00% |
| In top 50% IDACI Decile**** | 668 (49·81%) | 16 (53·33%) | 619 (49·71%) | 10 (50·00%) | 49 (51·04%) | 6 (60·00%) |
| Sixth Form | 840 (62·64%) | 22 (73·33%) | 782 (62·81%) | 15 (75·00%) | 57 (59·34%) | 7 (70·00%) |
| Selective Admissions | 73 (5·44%) | 4 (13·33%) | 60 (4·82%) | 1 (5·00%) | 13 (13·54%) | 3 (30·00%) |
| Faith School | 259 (19·31%) | 5 (16·67%) | 246 (19·76%) | 2 (10·00%) | 13 (13·54%) | 3 (30·00%) |
| Notes: All data are n (%) unless otherwise stated. *EAL = English as an Additional Language **FSM = Free School Meals ***SEN = Special Educational Needs ****Top 50% of Income Deprivation Affecting Children Index (IDACI) Deciles = Deciles 1 to 5 (those in the most deprived deciles) | | | | | | |

# Supplementary Table 3: Time current smartphone policies have been in place (Randhawa et al., 2024)

| **Current smartphone policy has been in place for:** | **Total Schools**  **(n=30)** | **Restrictive Schools (n=20)** | **Permissive Schools (n=10)** |
| --- | --- | --- | --- |
| More than 5 years | 6 | 3 | 3 |
| 2-5 years | 5 | 3 | 2 |
| Less than 2 years | 13 | 10 | 3 |
| Unknown/missing | 6 | 4 | 2 |

# Supplementary Table 4: Exploration of Interaction Effects Between Phone Policy and school Income Deprivation Affecting Children Index (IDACI), pupil Sex, pupil ethnicity, and pupil Year Group

|  | **Mental Wellbeing (WMWBS)**  **Coefficient (95% CI, p value)** | | **Smartphone Time - In School (hrs)**  **Coefficient (95% CI, p value)** | **Smartphone Time – Weekday**  **(hrs)**  **Coefficient (95% CI, p value)** | | **Smartphone Time – Weekend Day (hrs)**  **Coefficient (95% CI, p value)** | **Smartphone Time – Week**  **(hrs)**  **Coefficient (95% CI, p value)** | | **Social Media Time - In School**  **(hrs)**  **Coefficient (95% CI, p value)** | **Smartphone Time – Weekday**  **(hrs)**  **Coefficient (95% CI, p value)** | | **Smartphone Time – Weekend Day (hrs)**  **Coefficient (95% CI, p value)** | **Social Media Time – Week**  **(hrs)**  **Coefficient (95% CI, p value)** |
| --- | --- | --- | --- | --- | --- | --- | --- | --- | --- | --- | --- | --- | --- |
| **Socio-economic** | |  | | |  | | |  | | |  | | |
| Phone Policy Restrictive: IDACI | 0·29 (-0·4 to 0·94, p=0·51) | | 0·09 (-0·01 to 0·19, p=0·16) | 0·08 (-0·14 to 0·3, p=0·59) | | 0·05 (-0·35 to 0·45, p=0·85) | 0·63 (-1·20 to 2·55, p=0·60) | | 0·05 (-0·03 to 0·13, p=0·30) | 0·01 (-0·18 to 0·2, p=0·94) | | -0·08 (-0·4 to 0·25, p=0·69) | 0·01 (-1·42 to 1·52, p=0·99) |
| **Sex** | |  | | |  | | |  | | |  | | |
| Phone Policy Restrictive: Male | 2·00 (-0·28 to 4·05, p=0·075) | | 0·18 (-0·19 to 0·55, p=0·35) | -0·20 (-0·95 to 0·47, p=0·58) | | -0·48 (-1·50 to 0·45, p=0·34) | -1·48 (-6·93 to 3·47, p=0·58) | | 0·20 (-0·13 to 0·50, p=0·22) | -0·51 (-1·20 to 0·14, p=0·15) | | -0·72 (-1·71 to 0·12, p=0·13) | -3·62 (-8·81 to 0·85, p=0·15) |
| Phone Policy Restrictive: Prefer not to say | 8·06 (-1·13 to 17·05, p=0·084) | | 0·88 (-0·98 to 2·67, p=0·35) | -0·64 (-3·84 to 2·60, p=0·70) | | -1·72 (-6·05 to 2·59, p=0·43) | -6·05 (-29·52 to 17·33, p=0·61) | | 0·97 (-0·65 to 2·47, p=0·23) | -1·29 (-4·29 to 1·84, p=0·41) | | -3·73 (-7·80 to 0·37, p=0·074) | -13·89 (-36·08 to 8·15, p=0·22) |
| **Ethnicity** |  | |  |  | |  |  | |  |  | |  |  |
| Phone Policy Restrictive: Asian/Asian British | 1·07 (-1·68 to 3·98, p=0·47) | | 0·05 (-0·46 to 0·51, p=0·86) | -0·00 (-1·06 to 0·85, p > 0·99) | | -0·45 (-1·79 to 0·79, p=0·50) | -0·28 (-7·74 to 6·20, p=0·94) | | 0·07 (-0·36 to 0·46, p=0·75) | -0·53 (-1·39 to 0·36, p=0·25) | | -0·98 (-2·19 to 0·22, p=0·12) | -3·94 (-10·44 to 2·22, p=0·24) |
| Phone Policy Restrictive: Black/African/Caribbean | -4·27 (-9·69 to 1·21, p=0·13) | | 0·68 (-0·32 to 1·66, p=0·19) | 2·81 (0·83 to 4·50, p=0·0032) | | 2·81 (0·26 to 5·23, p=0·029) | 18·65 (4·92 to 31·38, p=0·0066) | | 0·25 (-0·62 to 1·07, p=0·57) | 2·03 (0·22 to 3·69, p=0·023) | | 2·38 (0·04 to 4·69, p=0·048) | 14·06 (1·52 to 26·10, p=0·028) |
| Phone Policy Restrictive: Mixed/Multiple | -2·13 (-6·51 to 2·19, p=0·34) | | 0·34 (-0·48 to 1·05, p=0·39) | 0·81 (-0·62 to 2·28, p=0·28) | | 2·65 (0·71 to 4·63, p=0·0086) | 8·52 (-1·80 to 19·08, p=0·11) | | 0·15 (-0·54 to 0·77, p=0·66) | 1·22 (-0·14 to 2·63, p=0·087) | | 2·67 (0·88 to 4·58, p=0·0050) | 10·50 (1·11 to 20·76, p=0·038) |
| Phone Policy Restrictive: Other ethnic group/I would rather not say/Missing | 4·58 (-0·56 to 9·93, p=0·090) | | 0·10 (-0·81 to 1·01, p=0·83) | -0·75 (-2·56 to 0·93, p=0·40) | | -1·67 (-4·03 to 0·66, p=0·17) | -7·29 (-19·99 to 5·00, p=0·26) | | 0·11 (-0·66 to 0·89, p=0·78) | -1·27 (-2·96 to 0·35, p=0·14) | | -3·47 (-5·66 to -1·26, p=0·0022) | -13·57 (-25·36 to -1·87, p=0·025) |
| **Year Group** |  | |  |  | |  |  | |  |  | |  |  |
| Phone Policy Restrictive: Year 10 | -0·61 (-3·06 to 1·87, p=0·65) | | -0·27 (-0·63 to 0·07, p=0·13) | 0·21 (-0·49 to 0·87, p=0·54) | | 0·04 (-0·86 to 0·93, p=0·93) | 0·54 (-4·31 to 5·21, p=0·83) | | -0·02 (-0·31 to 0·29, p=0·92) | 0·18 (-0·54 to 0·85, p=0·66) | | 0·42 (-0·48 to 1·27, p=0·36) | 1·06 (-4·22 to 5·95, p=0·69) |

**Notes:** Interaction terms included in the adjusted mixed effects regression models. School ID and Year group were included as random effects in all models. Other fixed effects variables included were: restrictive vs permissive phone policy, %Pupils with English as an Additional Language (EAL), %Pupils with Special Educational Needs (SEN), %Pupils eligible for (FSM), school size, school Income Deprivation Affecting Children Index (IDACI), school religious affiliation, school admissions policy, school co-education status, month of measurement, pupil sex, and pupil ethnicity,

# Supplementary Table 5 Adjusted Mean Differences in Mental Wellbeing by School Phone Policy (restrictive vs permissive phone policy group): Sensitivity Analysis

| **Variable** | **Mental Wellbeing: mean of two WEMWBS measures**  **(95% CI, p value) N=1212** | **Mental Wellbeing: first WEMWBS measure**  **(95% CI, p value) N=1207** |
| --- | --- | --- |
| Phone Policy: Restrictive | -0·48 (-2·05 to 1·06, p=0·62) | -1·15 (-2·72 to 0·42, p=0·19) |
| Admissions: Selective | -0·70 (-6·16 to 4·75, p=0·84) | -0·28 (-5·68 to 5·29, p=0·93) |
| %EAL | 0·03 (-0·03 to 0·10, p=0·42) | -0·01 (-0·08 to 0·06, p=0·79) |
| Ethnicity: Asian/Asian British | 0·42 (-0·96 to 2·01, p=0·58) | 0·76 (-0·80 to 2·32, p=0·35) |
| Ethnicity: Black/Black British/African/Caribbean | 0·93 (-1·28 to 3·22, p=0·42) | 0·44 (-1·87 to 2·89, p=0·72) |
| Ethnicity: Mixed/multiple | -2·62 (-4·77 to -0·45, p=0·019) | -3·23 (-5·57 to -1.00, p=0·0059) |
| Ethnicity: Other ethnic group/I would rather not say/Missing | -0·12 (-2·55 to 2·41, p=0·92) | -0·42 (-3·01 to 2·22, p=0·76) |
| %FSM | -0·08 (-0·27 to 0·12, p=0·53) | 0·02 (-0·17 to 0·22, p=0·83) |
| Gender: Male | 4·61 (3·57 to 5·57, p<0·0001) | 4·95 (3·86 to 5·97, p<0·0001) |
| Gender: Prefer not to say | -3·49 (-7·82 to 0·87, p=0·12) | -1·97 (-6·54 to 2·63, p=0·40) |
| IDACI | 0·14 (-0·23 to 0·51, p=0·53) | 0·14 (-0·23 to 0·51, p=0·49) |
| Religious Affiliation: Secular | -1·47 (-3·37 to 0·47, p=0·23) | -1·07 (-3·00 to 0·85, p=0·32) |
| School session: Summer | 0·06 (-1·37 to 1·51, p=0·94) | -0·47 (-1·91 to 0·98, p=0·56) |
| School Size | 0.00 (-0·00 to 0·00, p=0·94) | 0·00 (-0·00 to 0·00, p=0·26) |
| School Type: Single Sex | 1·23 (-3·36 to 5·89, p=0·67) | 0·91 (-3·78 to 5·51, p=0·73) |
| %SEN | -14·72 (-29·75 to 0·77, p=0·13) | -14·26 (-29·52 to 1·25, p=0·10) |

**Notes:** %EAL = %Pupils with English as an Additional Language. %FSM = %Pupils eligible for Free School Meals. IDACI = Income Deprivation Affecting Children Index. %SEN = %Pupils with Special Educational Needs.

# Supplementary Figure 5: The Distribution of Smartphone and Social Media Use Duration (hrs) in School in Permissive and Restrictive Schools


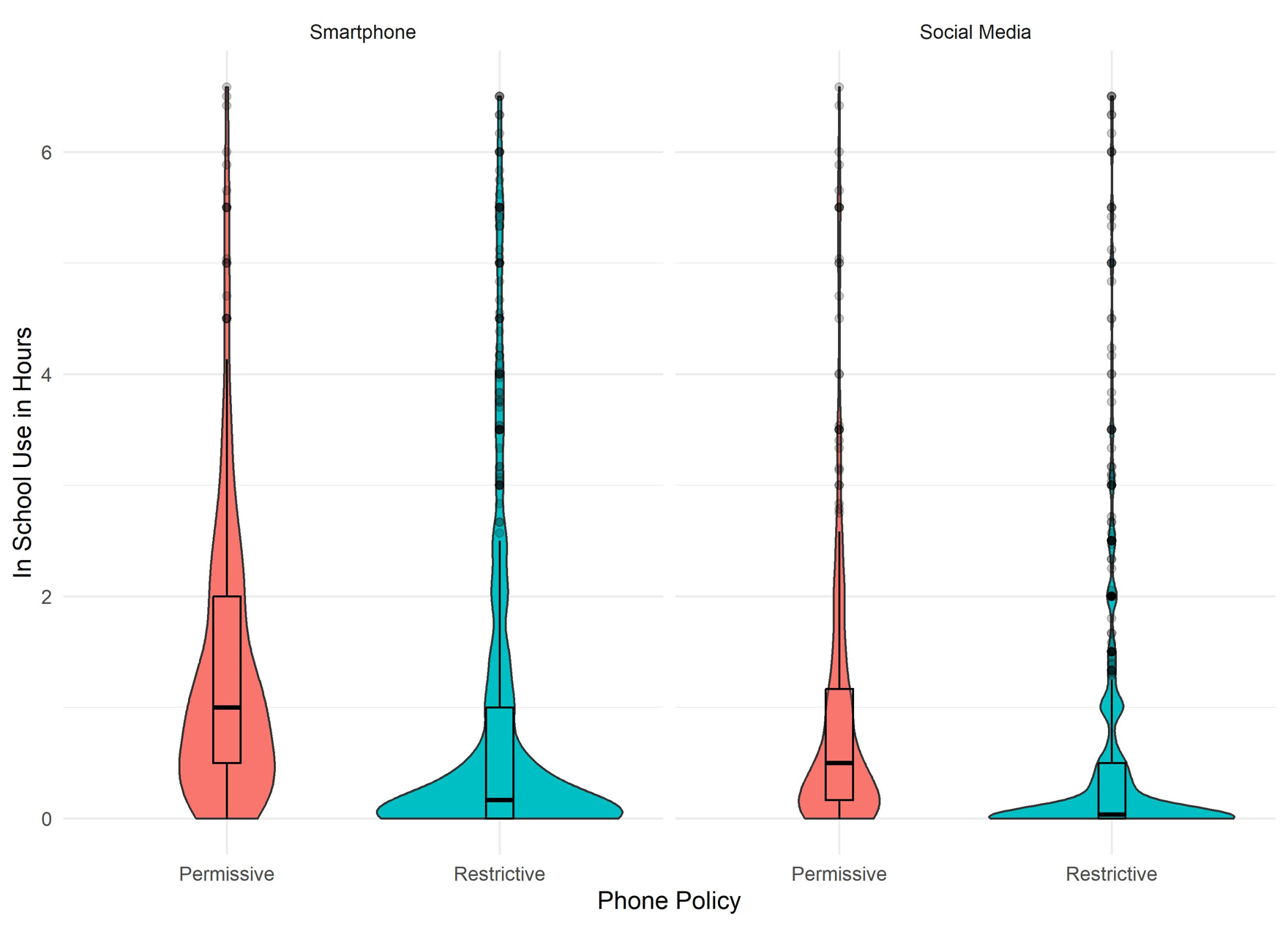


# Supplementary Table 6: Adjusted Mean Differences in Outcomes by School Phone Policy (restrictive vs permissive phone policy group [reference] compared with restrictive inaccessible school phone policies vs permissive phone policy group [reference]): Sensitivity Analysis

| **Variable** | **Adjusted Mean Difference**  **(95% CI, p value)**  **restrictive school policies (Schools=20)** | **Adjusted Mean Difference**  **(95% CI, p value) restrictive school policies**  **where phones are inaccessible (Schools=4)** |
| --- | --- | --- |
| Mental Wellbeing (WEMWBS) | -0·48 (-2·05 to 1·06, p=0·62) | -0·23 (-2·65 to 2·24, p=0·89) |
| Anxiety (GAD-7) | 0·1 (-0·76 to 0·97, p=0·84) | -0·32 (-1·73 to 1·05, p=0·72) |
| Depression (PHQ-9) | -0·04 (-0·98 to 0·92, p=0·94) | -0·21 (-1·86 to 1·33, p=0·83) |
| Problematic Use (PSMU) | 0·50 (-2·6 to 3·53, p=0·80) | -1·79 (-7·13 to 3·60, p=0·60) |
| Sleep Duration (mins) | 1·27 (-8·67 to 10·12, p=0·82) | -6·27 (-23·15 to 8·34, p=0·69) |
| Sleep Efficiency (%) | 0·40 (-0·83 to 1·60, p=0·61) | 0·58 (-0·96 to 2·65, p=0·77) |
| Sleep Window | 0·02 (-0·25 to 0·27, p=0·92) | -0·15 (-0·58 to 0·23, p=0·73) |
| Time of Falling Asleep | -0·18 (-0·42 to 0·07, p=0·21) | -0·34 (-0·71 to 0·03, p=0·32) |
| Wake Up Time | -0·22 (-0·56 to 0·12, p=0·30) | -0·47 (-0·93 to -0·04, p=0·37) |
| Moderate-to-Vigorous PA (mins) | 1·78 (-2·59 to 6·09, p=0·46) | 1·99 (-5·30 to 9·28, p=0·67) |
| Average Daily Acceleration (mg) | 1·14 (-1·6 to 3·82, p=0·45) | 0·29 (-4·27 to 4·78, p=0·92) |
| Attainment English=below target* | 1·45 (0·85 to 2·47, p=0·18) | 1·84 (0·95 to 3·57, p=0·070) |
| Attainment Maths=below target* | 1·01 (0·45 to 2·27, p=0·98) | 2·78 (1·35 to 5·72, p=0·0056) |
| Disruptiveness (PBQ) | 0·06 (-0·57 to 0·68, p=0·88) | 1·42 (0·18 to 2·6, p=0·28) |
| Conformity (SMUM) | -0·24 (-0·81 to 0·30, p=0·49) | -0·36 (-1·33 to 0·61, p=0·50) |
| Coping (SMUM) | 0·1 (-0·53 to 0·72, p=0·77) | -0·58 (-1·74 to 0·57, p=0·33) |
| Enhancement (SMUM) | -0·12 (-0·72 to 0·49, p=0·74) | -0·49 (-1·6 to 0·66, p=0·55) |
| Social (SMUM) | -0·31 (-0·95 to 0·36, p=0·45) | -0·19 (-1·38 to 1·04, p=0·85) |
| Screen Time - In School (hrs)** | -0.70 ( -0·91 to -0·49, p < 0·0001) | -0·72 ( -1·070 to -0·38, p < 0·0001) |
| Screen Time – Weekday (hrs) | 0·01 (-0·49 to 0·54, p=0·96) | -0·46 (-1·21 to 0·36, p=0·56) |
| Screen Time – Weekend Day (hrs) | 0·58 (-0·32 to 1·50, p=0·31) | 0·09 (-0·8 to 1·11, p=0·92) |
| Screen Time – Week (hrs) | 1·46 (-2·82 to 5·85, p=0·59) | -1·94 (-7·54 to 4·17, p=0·74) |
| Social Media – In School (hrs)** | -0.83 (-1·08 to -0·57, p < 0·0001) | -0·74 (-1·18 to -0·29, p = 0·0011) |
| Social Media – Weekday (hrs) | 0·25 (-0·18 to 0·69, p=0·33) | -0·26 (-0·94 to 0·44, p=0·53) |
| Social Media – Weekend Day (hrs) | 0·61 (-0·12 to 1·36, p=0·19) | 0.00 (-0·86 to 0·97, p > 0·99) |
| Social Media – Week (hrs) | 2·66 (-0·67 to 6·08, p=0·22) | -1·11 (-5·84 to 4·03, p=0·77) |

**Notes:** Wellbeing reported as WEMWBS score. Anxiety reported as GAD-7 score. Depression reported as PAQ-9 score. Addictive Social Media Use reported as PSMU score. Sleep Duration (hrs). Sleep Efficiency (%). Physical Activity (PA). Moderate-to-Vigorous PA (mins). Average Daily Acceleration (mg). Classroom Disruptiveness reported as PBQ score. *Mixed effects logistic regression models; odd ratios reported for attainment below target vs attainment on or above target. **Due to the extreme distribution of in school phone and social media time when looking at a smaller group of schools that required students lock their phones away, we had to use a negative binomial model without school ID entered as a random effect for this sensitivity analysis. Smartphone and Social Media Use Duration reported in hrs. School ID was included as a random effect in all models. Year group was included as a random effect in all models except for the unadjusted Attainment models. Fixed effects variables included in the adjusted models at the individual-level were: sex, and ethnicity, and at the school-level were: %Pupils with English as an Additional Language (EAL), %Pupils with Special Educational Needs (SEN), %Pupils eligible for (FSM), school size, school Income Deprivation Affecting Children Index (IDACI), school religious affiliation, school admissions policy, school co-education status, and month of measurement. Restrictive school policies where phones are inaccessible represent 4 schools with policies, including: phones must be kept in lockers (n=1), phones must be kept in a pouch (n=1), phones must be handed into the school (n=1) and phones are not allowed onto school premises (n=1).

# Supplementary Table 7. Participant-level Outcome Data Summarised by School Group

|  | **Permissive**, N = 407 | **Restrictive**, N = 820 |
| --- | --- | --- |
| **Mental Health and Wellbeing** |  |  |
| **Wellbeing** Mean (SD)  Missing | 48 (9)  1 (0·2%) | 46 (9)  3 (0·4%) |
| **Wellbeing Group** N (%) |  |  |
| High | 38 (9·36%) | 55 (6·73%) |
| Medium | 255 (62·81%) | 487 (59·61%) |
| Low | 113 (27·83%) | 275 (33·66%) |
| **Anxiety** Mean (SD)  Missing | 6·5 (5·1)  6 (1·5%) | 7·2 (5·4)  7 (0·9%) |
| **Anxiety Group** N (%) |  |  |
| Minimal anxiety | 166 (41·40%) | 317 (38·99%) |
| Mild anxiety | 125 (31·17%) | 244 (30·01%) |
| Moderate anxiety | 80 (19·95%) | 155 (19·07%) |
| Severe anxiety | 30 (7·48%) | 97 (11·93%) |
| **Depression** Mean (SD)  Missing | 7·0 (5·7)  8 (2·0%) | 7·7 (6·2)  12 (1·5%) |
| **Depression Group** N (%) |  |  |
| Minimal depression | 168 (42·11%) | 338 (41·83%) |
| Mild depression | 123 (30·83%) | 186 (23·02%) |
| Moderate depression | 60 (15·04%) | 151 (18·69%) |
| Moderately severe depression | 35 (8·77%) | 91 (11·26%) |
| Severe depression | 13 (3·26%) | 42 (5·20%) |
| **Problematic Social Media Use** Median (IQR)  Missing | 51 (34, 65)13 (3·2%) | 57 (36, 68)  15 (1·8%) |
| **Sleep and Physical Activity (PA)** Mean (SD) |  |  |
| Sleep Duration (hours)  Missing | 7·26 (0·73)  170 (42·0%) | 7·34 (0·77)  290 (35·0%) |
| Sleep Efficiency (%)  Missing | 0·85 (0·05)  170 (42·0%) | 0·85 (0·05)  290 (35·0%) |
| Moderate-to-Vigorous PA (mins/day)  Missing | 31 (16)  155 (38·0%) | 30 (15)  278 (34·0%) |
| Average Daily Acceleration (m*g*)  Missing | 34 (10)  155 (38·0%) | 33 (10)  278 (34·0%) |
| **Attainment** N (%) |  |  |
| **English Group** |  |  |
| Above target | 60 (15·35%) | 126 (17·43%) |
| On target | 207 (52·94%) | 290 (40·11%) |
| Below target  Missing | 124 (31·71%)  16 (3·9%) | 307 (42·46%)  97 (12·0%) |
| **Maths Group** |  |  |
| Above target | 71 (17·84%) | 161 (22·27%) |
| On target | 191 (47·99%) | 271 (37·48%) |
| Below target  Missing | 136 (34·17%)  9 (2·2%) | 291 (40·25%)  97 (12·0%) |
| **Classroom Behaviour** Mean (SD) |  |  |
| Disruptiveness  Missing | 1·44 (2·31)  48 (12·0%) | 1·32 (2·23)  72 (8·8%) |
| **Social Media Use Motives** Median (IQR) |  |  |
| SMUM – Coping  Missing | 10·0 (7·0, 13·0)  12 (2·9%) | 11·0 (8·0, 14·0)  15 (1·8%) |
| SMUM – Conformity  Missing | 5·00 (4·00, 8·00)  12 (2·9%) | 5·00 (4·00, 8·00)  11 (1·3%) |
| SMUM – Enhancement  Missing | 13·0 (10·0, 16·0)  13 (3·2%) | 13·0 (10·0, 16·0)  11 (1·3%) |
| SMUM – Social  Missing | 14·0 (11·0, 16·0)  12 (2·9%) | 13·0 (10·0, 16·0)  11 11 (1·3%) |
| **Self-reported smartphone and social media use duration in hours** Median (IQR) [10^th^ and 90^th^ percentile] | | |
| Smartphone Time - In School  Missing | 1·00 (0·50, 2·00) [0·17, 3·00]  41(10%) | 0·17 (0·00, 1·00) [0·00, 3·00]  78 (9·5%) |
| Smartphone Time – Weekday  Missing | 4·00 (2·50, 5·76) [1·71, 7·72]  21 (5·2%) | 4·50 (3·00, 6·50) [1·75, 8·70]  37 (4·5%) |
| Smartphone Time – Weekend Day | 5·0 (3·4, 7·5) [2·0, 10·3]  24 (5·9%) | 6·0 (4·0, 9·0) [2·5, 12·0]  47 (5·7%) |
| Smartphone Time – Week  Missing | 31 (20, 44) [14, 58]  29 (7·1%) | 35 (23, 50) [15, 66]  55 (6·7%) |
| Social Media Time - In School  Missing | 0·50 (0·17, 1·17) [0·00, 2·50]  41 (10·0%) | 0·03 (0·00, 0·50) [0·00, 2·00]  78 (9·5%) |
| Social Media Time – Weekday  Missing | 2·54 (1·25, 4·50) [0·50, 6·00]  21 (5·2%) | 3·17 (1·67, 5·03) [1·00, 7·50]  37 (4·5%) |
| Social Media Time – Weekend Day  Missing | 3·3 (1·8, 5·5) [1·0, 8·0]  24 (5·9%) | 4·3 (2·3, 7·0) [1·0, 10·0]  47 (5·7%) |
| Social Media Time – Week  Missing | 20 (10, 32) [6, 44]  29 (7·1%) | 25 (14, 39) [7, 56]  55 (6·7%) |

**Notes:** Data are n (%) unless otherwise stated. Wellbeing reported as WEMWBS score. Anxiety reported as GAD-7 score. Depression reported as PAQ-9 score. Problematic Social Media Use reported as PSMU score. Classroom Disruptiveness reported as PBQ score. Social Media Use Motives reported as SMUM score for coping, conformity, enhancement and social. See Supplementary File Table 1 for scoring/unit of measurement for each outcome measure.

# Supplementary Table 8 Adjusted Mean Differences in Outcomes by School Phone Policy (restrictive vs permissive phone policy group) controlling for weekend day smartphone time: Sensitivity Analysis

| **Variable** | **Adjusted Mean Difference**  **(95% CI, p value)**  **restrictive school policies (n=20)** | **Adjusted Mean Difference**  **(95% CI, p value) controlling for**  **weekend smartphone time** |
| --- | --- | --- |
| Mental Wellbeing (WEMWBS) | -0·48 (-2·05 to 1·06, p=0·62) | -0·45 (-1·98 to 1·06, p=0·62) |
| Anxiety (GAD-7) | 0·1 (-0·76 to 0·97, p=0·84) | 0·14 (-0·67 to 0·97, p=0·77) |
| Depression (PHQ-9) | -0·04 (-0·98 to 0·92, p=0·94) | -0·01 (-0·93 to 0·93, p=0·99) |
| Problematic Use (PSMU) | 0·50 (-2·6 to 3·53, p=0·80) | -0·53 (-3·47 to 2·34, p=0·76) |
| Sleep Duration (mins) | 1·27 (-8·67 to 10·12, p=0·82) | 2·01 (-7·81 to 10·39, p=0·72) |
| Sleep Efficiency (%) | 0·40 (-0·83 to 1·60, p=0·61) | 0·50 (-0·78 to 1·76, p=0·55) |
| Sleep Window | 0·02 (-0·25 to 0·27, p=0·92) | 0·02 (-0·24 to 0·26, p=0·91) |
| Time of Falling Asleep | -0·18 (-0·42 to 0·07, p=0·21) | -0·24 (-0·48 to 0·02, p=0·11) |
| Wake Up Time | -0·22 (-0·56 to 0·12, p=0·30) | -0·28 (-0·63 to 0·08, p=0·22) |
| Moderate-to-Vigorous PA (mins) | 1·78 (-2·59 to 6·09, p=0·46) | 1·64 (-2·79 to 6·03, p=0·51) |
| Average Daily Acceleration (mg) | 1·14 (-1·6 to 3·82, p=0·45) | 1·16 (-1·67 to 3·94, p=0·46) |
| Attainment English=below target* | 1·45 (0·85 to 2·47, p=0·18) | 1·47 (0·84 to 2·54, p=0·17) |
| Attainment Maths=below target* | 1·01 (0·45 to 2·27, p=0·98) | 1·01 (0·46 to 2·22, p=0·97) |
| Disruptiveness (PBQ) | 0·06 (-0·57 to 0·68, p=0·88) | 0·05 (-0·49 to 0·58, p=0·89) |
| Conformity (SMUM) | -0·24 (-0·81 to 0·30, p=0·49) | -0·18 (-0·75 to 0·38, p=0·60) |
| Coping (SMUM) | 0·1 (-0·53 to 0·72, p=0·77) | -0·05 (-0·67 to 0·56, p=0·87) |
| Enhancement (SMUM) | -0·12 (-0·72 to 0·49, p=0·74) | -0·19 (-0·78 to 0·43, p=0·59) |
| Social (SMUM) | -0·31 (-0·95 to 0·36, p=0·45) | -0·34 (-0·99 to 0·33, p=0·39) |
| Screen Time - In School (hrs) | -0·67 (-0·92 to -0·43, p=0·00024) | -0·75 (-1·00 to -0·50, p = 0·00010) |
| Screen Time – Weekday (hrs) | 0·01 (-0·49 to 0·54, p=0·96) | -0·25 (-0·57 to 0·07, p=0·16826) |
| Screen Time – Week (hrs) | 1·46 (-2·82 to 5·85, p=0·59) | -1·26 (-2·84 to 0·35, p=0·17) |
| Social Media – In School (hrs) | -0·54 (-0·74 to -0·36, p=0·00018) | -0·61 (-0·8 to -0·42, p<0·0001) |
| Social Media – Weekday (hrs) | 0·25 (-0·18 to 0·69, p=0·33) | 0·01 (-0·31 to 0·34, p=0·94) |
| Social Media - Week (hrs) | 2·66 (-0·67 to 6·08, p=0·22) | 0·41 (-1·54 to 2·37, p=0·73) |

**Notes:** **:** Wellbeing reported as WEMWBS score. Anxiety reported as GAD-7 score. Depression reported as PAQ-9 score. Addictive Social Media Use reported as PSMU score. Sleep Duration (hrs). Sleep Efficiency (%). Physical Activity (PA). Moderate-to-Vigorous PA (mins). Average Daily Acceleration (mg). Classroom Disruptiveness reported as PBQ score. *Mixed effects logistic regression models; odd ratios reported for attainment below target vs attainment on or above target. Smartphone and Social Media Use Duration reported in hrs. School ID was included as a random effect in all models. Year group was included as a random effect in all models except for the unadjusted Attainment models. Fixed effects variables included in the adjusted models at the individual-level were: sex, and ethnicity, and at the school-level were: %Pupils with English as an Additional Language (EAL), %Pupils with Special Educational Needs (SEN), %Pupils eligible for (FSM), school size, school Income Deprivation Affecting Children Index (IDACI), school religious affiliation, school admissions policy, school co-education status, and month of measurement.

Supplementary Figure 5: Pearson Correlation between Self-Report Smartphone Time In-School, across the School Day and for a Weekend Day
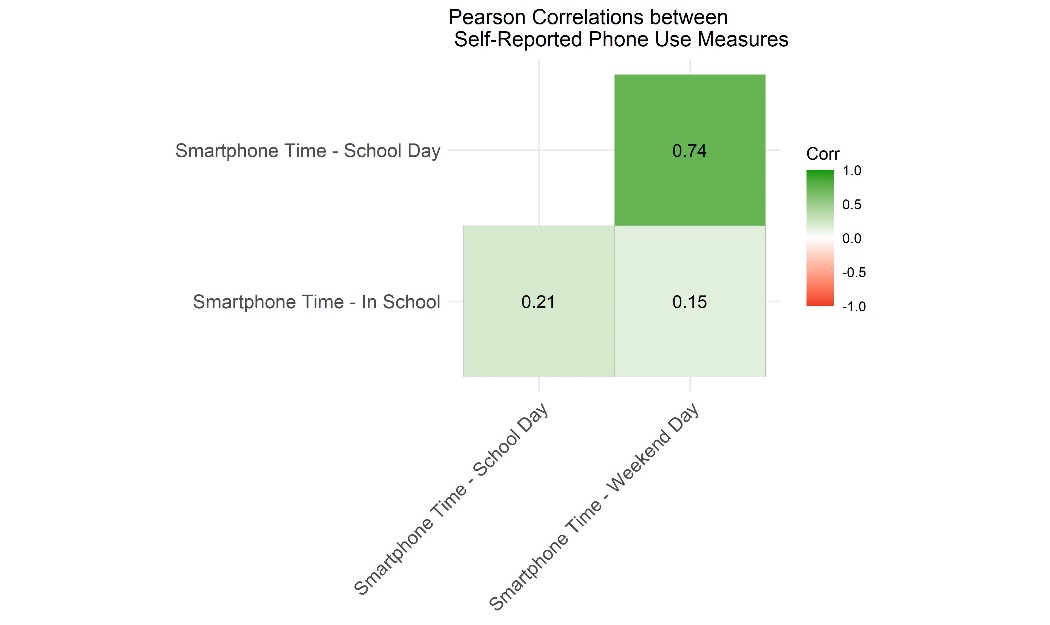


# Supplementary Figure 6: Pearson Correlation between Self-Report Social Media Time In-School, across the School Day and for a Weekend Day


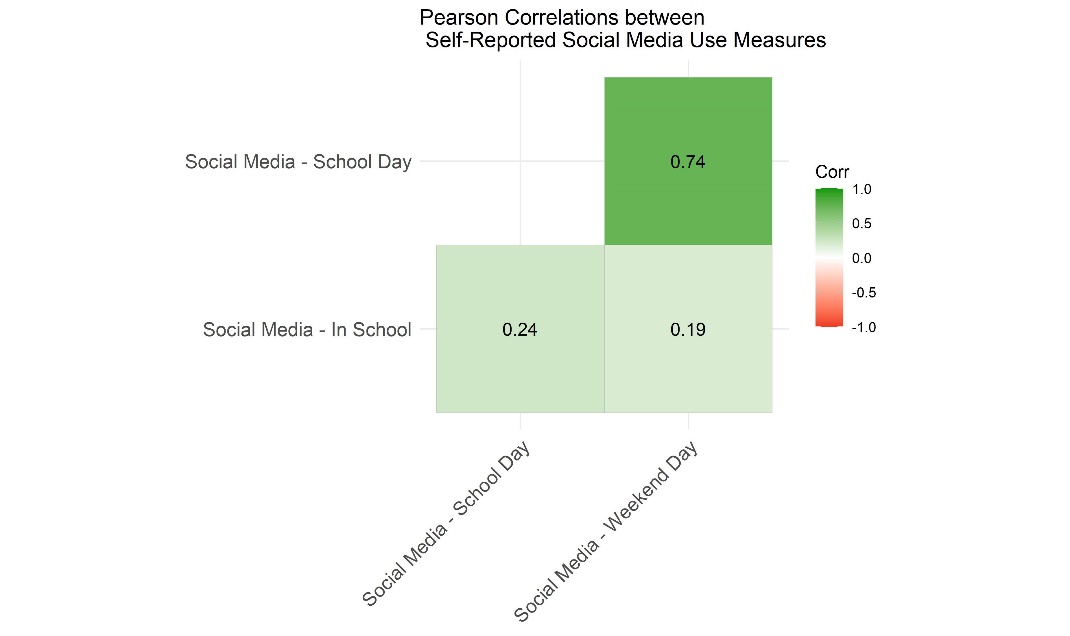


Supplementary Figure 7: Proportion of pupils reporting higher social media time than phone screen time from self-reported phone data between permissive and restrictive schools
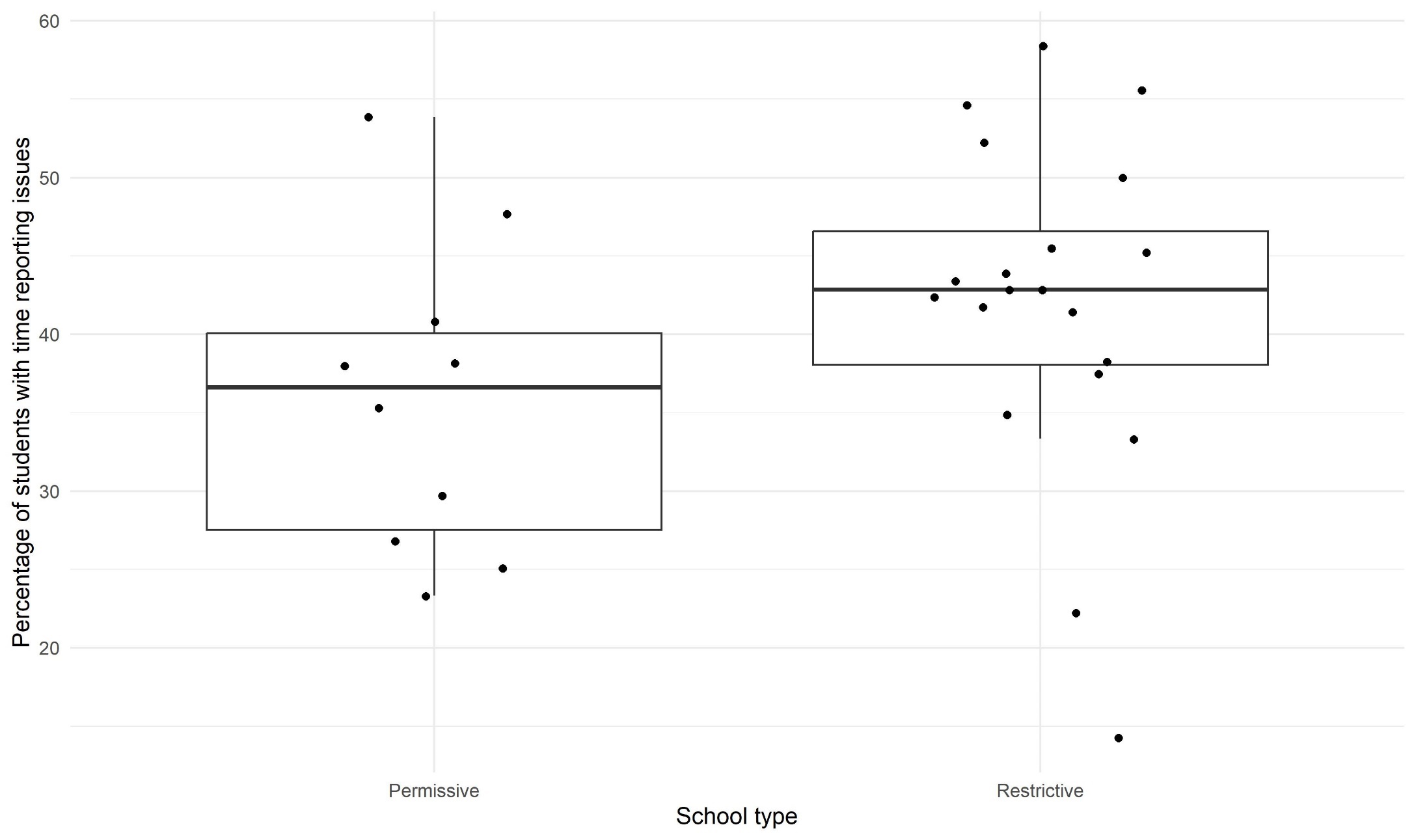


# Summary of Ethical Procedures for Opt Out Consent

Ethics approval This study involves human participants and full ethical approval was obtained from the University of Birmingham’s Science Technology, Engineering and Mathematics Research Ethics Committee on 8 July 2022 (ERN_22-0723).

The procedures for opt out were co-produced as part of the PPI process, and were also discussed and approved by the study Data Monitoring and Ethics Committee (DMEC) and the Study Steering Committee (SSC) before receiving final sign off by our sponsor and funder – The NIHR. The ethical procedures and subsequent safeguarding procedures for this study were highly commended by our SSC and were recommended to be published in the BMJ Open.

*Randhawa A, Wood G, Michail M, et al. Safeguarding in adolescent mental health research: navigating dilemmas and developing procedures. BMJ Open 2024;14:e076700. doi:10.1136/ bmjopen-2023-076700*

We have outlined our ethical procedures below.

To ensure all information and opt out material reached parents, we took the following steps, and these are reported in our ethical approval form.

1. A meeting was held with the school liaison member of staff to make arrangements for data collection in the school. In this step we identified the most effective pathways for communication with parents to ensure that the information on opt out consent (see below sections) reached them, including, where necessary the translation of information into relevant languages, and the need to contact ‘responsible adult’ in care of the child (e.g. for looked after children). We also provided the school with a support package on mental health to help the school support pupil mental health, if any concerns were identified with pupils as part of the research. ***Our DMEC and SSC for the study identified this as an important step to ensure information reaches parents, and schools are provided with additional information on mental health in further support of school’s already established practices***
2. Pupils and teachers were recruited from classes in year 8 (age 12-13) and year 10 (age 14-15) which were identified by the liaison member of staff.

Pupils of participating classes were shown a video about the study and were provided with a recruitment flyer during lesson time, that included links to the study website and pupil online privacy statement. The pupils had the opportunity to ask questions to their teachers and/or contact the research team by email

The liaison member of staff arranged for an information letter to be sent to parents/carers of potential participating pupils from the selected classes. The information letter included detailed information on the study procedures, what was requested of pupils and how the data were processed, and a video and link to the study website were provided to ensure the information was accessible. The information pack included an opt-out form, which parents were asked to complete if they did not wish for their child to participate. The information pack was sent to parents/carers in different formats (email, text, post, hard copy letters, text and via school website) and followed the schools typical processes for school-home communication to ensure the information reached parents/carers. Parents/carers had the opportunity to ask questions to the liaison member of staff and/or were provided with a contact email to discuss the study with the research team. ***Our PPI with youth, teachers and parents established that the most effective mechanism to ensure the information reaches parents was through email/text, and to ensure parents have the opportunity to respond via email and/or via the website. ***

Prior to data collection, the researchers checked with the school liaison member of staff via email or phone that:

- Information letters had been sent to parents a minimum of 7 days prior to data collection
- Identified any opt out consent reported to school (parents could return opt out consent by paper, text message, email or by completing an online survey).

This information was further cross-checked with the school liaison member of staff and the class teacher when the researchers visited the school and prior to any data collection.

Pupil Survey**
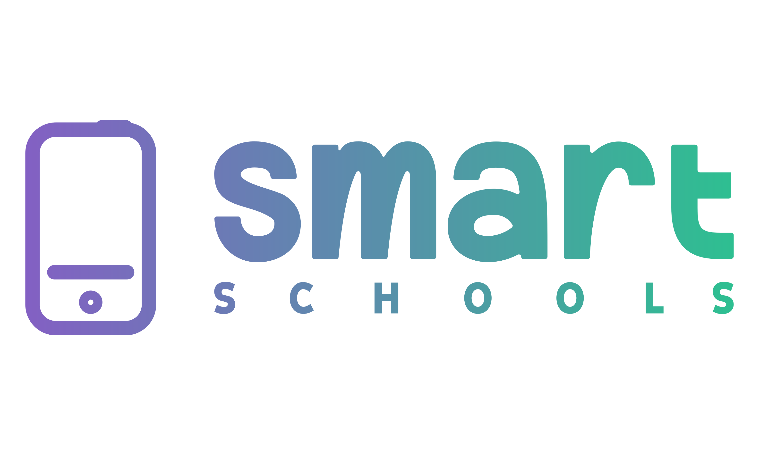

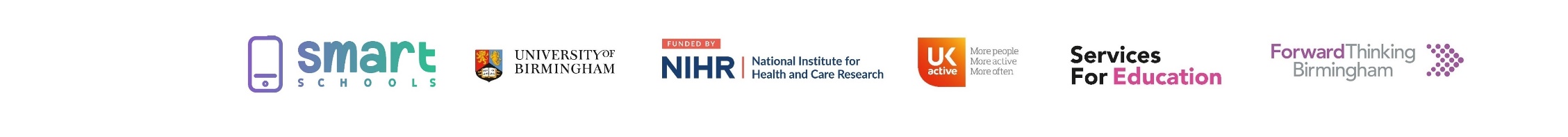
**

**SMART Schools Study: Smartphones, social Media and Adolescent mental wellbeing: the impact of school policies Restricting dayTime use**

This survey is organised into 9 sections and will ask you about:

1. Assent
2. Personal Details
3. Activity and Sleep
4. Mental Wellbeing
5. Anxiety and Depression
6. Health and Quality of Life
7. Motives and Reasons for using Social Media
8. Smartphone and Social Media use
9. Phone Task

You can stop completing the survey at any time, and if you stop completing you will not be affected in any way. If you want to stop completing the survey, please let your class teacher know and/or a member of the research team and they can provide you with an alternative task. If at any time you feel in distress from completing the survey, please inform your teacher or a member of the research team.

Your answers to this survey will be confidential. Only the researchers will know what you have said and *we will not share your individual responses with anyone outside of the research team*. However, we may alert a teacher in your school if your answers to questions about depression suggest that you may benefit from mental health support.

| SECTION 1: Pupil Assent |
| --- |

**Please read and tick the following before you start the survey:**

- I confirm that I have read and understood the Pupil Information Flyer dated 14/03/2023 (Version 6.0), and have had an opportunity to ask questions
- I understand that I will be asked to complete an online survey (up to 30 minutes), wear an activity monitor watch at home and school for 7 days and complete another survey (5 minutes), 4 weeks later
- I understand that I may be asked to take part in a focus group discussion
- I understand that my participation is voluntary and that I am free to withdraw (remove myself from the study) at any time without my rights being affected
- I understand that I can ask for my information to be deleted up to 4 weeks after I have taken part in the study, and that if I want to withdraw myself from the study, I should inform my class teacher and/or email the research team smartstudy@contacts.bham.ac.uk
- I understand that my answers will be kept confidential and only members of the research team will have access to my information.
- I understand that the exception to this is if I, or a member of my class, identifies a safeguarding concern. I understand that if this were to occur, that the researcher will inform a member of school staff so that they can help provide extra support.

**Do you want to take part in the SMART Schools Study?**

- Yes
- No

If you DO NOT wish to take part in the study, you can complete an alternative task that can be accessed here: Year 8: <https://bbc.in/3PgJLMP> Year 10: <https://bbc.in/3wnnww5>

**Would you like to be contacted in the future about taking part in further research with the University of Birmingham?**

- Yes
- No

**If yes, please insert email address__________________________________**

Please answer honestly. If you do not wish to answer certain questions you do not have to.

| SECTION 2: Personal Details |
| --- |

**Please enter your SMART ID here. This is the four digit number on the card in the pack provided:**

**­­­­_________________________________________________________________________**

This section asks you questions about your background.

1. **What is your year group?**

- Year 8
- Year 10

1. **What is your name?**
2. First name: _____________________________________
3. Surname: _______________________________________________
4. **Date of Birth** (DD/MM/YYYY) ______ / ________ / ___________
5. **What is your home postcode?** If you have more than one address, please use the one where you spend most of your time. *(Please write in full)* ________________________________
6. **Is the gender you identify with the same as your sex registered at birth?**

- Yes
- No

If no, please enter gender identity_________________________________________

- I would rather not say

1. **What is your sex?** *(Select one box only)*

- Female
- Male
- I would rather not say

1. **Please select one group from the list below that best describes you**:

**White**

- English / Welsh / Scottish / Northern Irish / British
- Irish
- Gypsy or Irish Traveller
- Any other White background *(please specify)*

________________________________________________________________

**Mixed / Multiple ethnic groups**

- White and Black Caribbean
- White and Black African
- White and Asian
- Any other Mixed / Multiple ethnic background *(please specify)* _______________________________________________________________

**Asian / Asian British**

- Indian
- Pakistani
- Bangladeshi
- Chinese
- Any other Asian background *(please specify)* ________________________________________________________________

**Black / African / Caribbean / Black British**

- African
- Caribbean
- Any other Black / African / Caribbean background *(please specify)*

**Other ethnic group**

- Arab
- Any other ethnic group *(please specify)*____________________________________
- I would rather not say

**Please enter your SMART ID again. This is the four digit number on the card in the pack provided:**

**_________________________________________________________________________**

| SECTION 3: Activity and Sleep |
| --- |

This section asks you about your physical activity and sleep behaviours.

1. **What is the main mode of transport you use to get to and from school?** *(Please select one option, i.e. the mode of transport that you use for the majority of the time)*

- Walk
- Car
- Bus
- Bike
- Scooter
- Electronic Scooter
- Other *(please specify)* _________________________________________________________

**9 a). On a typical school day, what time do you leave your house for school?**

___: __(am)

 **b). On a typical school day, what time do you arrive at school?** ____: ___(am)
 **c). On a typical school day, what time do you leave school?** ___: ___ (pm)
 **d). On a typical school day, what time do you arrive at your house** ____: ____ (pm)

- - - 1. **a). Do you take part in any clubs before school starts?**
- Yes
- No

**b). If yes, which of the following clubs do you take part in before school starts? (Please select all that apply and list all the days that you attend)**

- Band, orchestra or choir

Day(s) of the week:______________

- School play or school musical

Day(s) of the week:______________

- School yearbook, newspaper or magazine

Day(s) of the week:______________

- Volunteering or service activities

Day(s) of the week:______________

- Science club

Day(s) of the week:______________

- Science competitions

Day(s) of the week:______________

- Chess club

Day(s) of the week:______________

- Club with a focus on computers and ICT

Day(s) of the week:______________

- Art club/activities

Day(s) of the week:______________

- Sporting team/activities

Day(s) of the week:______________

- Other (please specify) _____________________________________________

Day(s) of the week:______________

- Other *(please specify)*_____________________________________________

Day(s) of the week:______________

- Other *(please specify)* _____________________________________________

Day(s) of the week:______________

- - - 1. **a). Do you take part in any clubs during your lunch break?**
- Yes
- No

**b). If Yes, which of the following clubs do you take part in during your lunch break? (Please select all that apply and list all the days that you attend)**

- Band, orchestra or choir

Day(s) of the week:______________

- School play or school musical

Day(s) of the week:______________

- School yearbook, newspaper or magazine

Day(s) of the week:______________

- Volunteering or service activities

Day(s) of the week:______________

- Science club

Day(s) of the week:______________

- Science competitions

Day(s) of the week:______________

- Chess club

Day(s) of the week:______________

- Club with a focus on computers and ICT

Day(s) of the week:______________

- Art club/activities

Day(s) of the week:______________

- Sporting team/activities

Day(s) of the week:______________

- Other *(please specify)* ________________________________________________

Day(s) of the week:______________

- Other *(please specify)* ________________________________________________

Day(s) of the week:______________

- Other *(please specify)* ________________________________________________

Day(s) of the week:______________

**12 a). Do you take part in any school clubs after school?**

- Yes
- No

**b). If yes, which of the following school clubs do you take part in after school? (Please select all that apply and list all the days that you attend)**

- Band, orchestra or choir

Day(s) of the week:______________

- School play or school musical

Day(s) of the week:______________

- School yearbook, newspaper or magazine

Day(s) of the week:______________

- Volunteering or service activities

Day(s) of the week:______________

- Science club

Day(s) of the week:______________

- Science competitions

Day(s) of the week:______________

- Chess club

Day(s) of the week:______________

- Club with a focus on computers and ICT

Day(s) of the week:______________

- Art club/activities

Day(s) of the week:______________

- Sporting team/activities

Day(s) of the week:______________

- Other *(please specify)* _________________________________________________

Day(s) of the week:______________

- Other *(please specify)* _________________________________________________

Day(s) of the week:______________

- Other *(please specify)*__________________________________________________

**13 a). Do you take part in any clubs after school that are not related to the school (e.g. dance, sports clubs, Scouts, Guides)**

- Yes
- No

**b). If yes, which of the following non-school related clubs do you take part in after school? (Please select all that apply and list all the days that you attend)**

- Youth clubs, scouts, girl guides or other organised activities

Day(s) of the week:______________

- Voluntary or community work

Day(s) of the week:______________

- Music

Day(s) of the week:______________

- Art

Day(s) of the week:______________

- Dance

Day(s) of the week:______________

- Sport

Day(s) of the week:______________

- Tutorials for school subjects

Day(s) of the week:______________

- Religious classes

Day(s) of the week:______________

- Other *(please specify)* __________________________________________________

Day(s) of the week:______________

- Other *(please specify)* __________________________________________________

Day(s) of the week:______________

**14 a). Do you take part in any clubs on a weekend?**

- Yes
- No

**b). If yes, which of the following clubs do you take part in on a weekend?** *(Please select all that apply)*

- Youth clubs, scouts, girl guides or other organised activities
- Voluntary or community work
- Music
- Art
- Dance
- Sport
- Tutorials for school subjects
- Religious classes
- Other *(please specify)* _________________________________________________
- Other *(please specify)*__________________________________________________

**How much time do you spend taking part in this club on a weekend?** *(e.g. 1 hour and 30 minutes)*

_______________________ hours _____________________ minutes

- - - 1. a). On a school day, what time do you normally wake up? ___: ___

b). When you have school the next day, what time do you normally go to sleep? __: __

c). On a weekend, what time do you normally wake up? ___: ___

d). When it is the weekend the next day, what time do you normally go to sleep? __: __

| SECTION 4: Mental Wellbeing |
| --- |

This section asks you questions about your mental wellbeing.

- 1. **Below are some statements about feelings and thoughts. Please tick the box that best describes your experience of each over the last 2 weeks** *(please select only one option for each statement)*

|  | None of the time | Rarely | Some of the time | Often | All of the time |
| --- | --- | --- | --- | --- | --- |
| a). I’ve been feeling optimistic about the future |  |  |  |  |  |
| b). I’ve been feeling useful |  |  |  |  |  |
| c). I’ve been feeling relaxed |  |  |  |  |  |
| d). I’ve been feeling interested in other people |  |  |  |  |  |
| e). I’ve had energy to spare |  |  |  |  |  |
| f). I’ve been dealing with problems well |  |  |  |  |  |
| g). I’ve been thinking clearly |  |  |  |  |  |
| h). I’ve been feeling good about myself |  |  |  |  |  |
| i). I’ve been feeling close to other people |  |  |  |  |  |
| j). I’ve been feeling confident |  |  |  |  |  |
| k). I’ve been able to make up my own mind about things |  |  |  |  |  |
| l). I’ve been feeling loved |  |  |  |  |  |
| m). I’ve been interested in new things |  |  |  |  |  |
| n). I’ve been feeling cheerful |  |  |  |  |  |

| SECTION 5: Anxiety and Depression |
| --- |

This section asks you questions about problems that can indicate anxiety and depression.

- 1. **Over the last two weeks, how often have you been bothered by any of the following problems** *(please select only one option for each problem)*

|  | Not at all | Several days | More than half the days | Nearly everyday |
| --- | --- | --- | --- | --- |
| a). Feeling nervous, anxious or on edge |  |  |  |  |
| b). Not being able to stop or control your worrying |  |  |  |  |
| c). Worrying too much about different things |  |  |  |  |
| d). Trouble relaxing |  |  |  |  |
| e). Being so restless that it’s hard to sit still |  |  |  |  |
| f). Becoming easily annoyed or irritable |  |  |  |  |
| g). Feeling afraid as if something awful might happen |  |  |  |  |

- 1. **Over the last two weeks, how often have you been bothered by any of the following problems** *(please select only one option for each problem)*

|  | Not at all | Several days | More than half the days | Nearly everyday |
| --- | --- | --- | --- | --- |
| a). Little interest or pleasure in doing things |  |  |  |  |
| b). Feeling down, depressed or hopeless |  |  |  |  |
| c). Trouble falling or staying asleep, or sleeping too much |  |  |  |  |
| d). Feeling tired or having little energy |  |  |  |  |
| e). Poor appetite or overeating |  |  |  |  |
| f). Feeling bad about yourself or that you’re a failure or have let yourself or your family down |  |  |  |  |
| g). Trouble concentrating on things, such as reading or watching TV |  |  |  |  |
| h). Moving or speaking so slowly that other people could have noticed? Or the opposite – being so fidgety or restless that you have been moving around a lot more than usual |  |  |  |  |
| i). Thoughts that you would be better off dead or of hurting yourself in some way |  |  |  |  |

| SECTION 6: Health and Quality of Life |
| --- |

This section asks you questions about your feelings and what we call “quality of life.”

- 1. **These following questions ask about how you are today. For each question, please read all the choices and decide which one is most like you today***. (Select only one box for each question)*

1. **Worried**

- I don’t feel worried today
- I feel a little bit worried today
- I feel a bit worried today
- I feel quite worried today
- I feel very worried today

1. **Sad**

- I don’t feel sad today
- I feel a little bit sad today
- I feel a bit sad today
- I feel quite sad today
- I feel very sad today

1. **Pain**

- I don’t have any pain today
- I have a little bit of pain today
- I have a bit of pain today
- I have quite a lot of pain today
- I have a lot of pain today

1. **Tired**

- I don’t feel tired today
- I feel a little bit tired today
- I feel a bit tired today
- I feel quite tired today
- I feel very tired today

1. **Annoyed**

- I don’t feel annoyed today
- I feel a little bit annoyed today
- I feel a bit annoyed today
- I feel quite annoyed today
- I feel very annoyed today

1. **School Work/Homework (such as reading, writing, doing lessons)**

- I have no problems with my schoolwork/homework today
- I have a few problems with my schoolwork/homework today
- I have some problems with my schoolwork/homework today
- I have many problems with my schoolwork/homework today
- I can’t do my schoolwork/homework today

1. **Sleep**

- Last night I had no problems sleeping
- Last night I had a few problems sleeping
- Last night I had some problems sleeping
- Last night I had many problems sleeping
- Last night I couldn’t sleep at all

1. **Daily routine (things like eating, having a bath/shower, getting dressed)**

- I have no problems with my daily routine today
- I have a few problems with my daily routine today
- I have some problems with my daily routine today
- I have many problems with my daily routine today
- I can’t do my daily routine today
  1. **Able to join in activities (things like playing out with your friends, doing sports, joining in things)**
- I can join in with any activities today
- I can join in with most activities today
- I can join in with some activities today
- I can join in with a few activities today
- I can join in with no activities today

| SECTION 7: Motives and Reasons for Using Social Media |
| --- |

This section is about your uses of social media and your reasons for using it. When answering these questions, we would like you to think of “social media” as any app on your smartphone that you use to interact with other people and that involves a profile to set up, for example; YouTube, TikTok, Instagram, Snapchat. When answering questions about social media please do not include apps or platforms for entertainment/watching TV and gaming, such as; Netflix, Xbox chat and PlayStation hangout.

- 1. **The following question lists different reasons for using social media. Thinking of all of the times you have been on social media in the last 12 months, please rate on a scale from *‘never’* to ‘*always*’, how often you have logged onto social media for each of these following reasons** (*select only one answer for each statement)*

|  | Never | Almost Never | Occasionally | Almost always | Always |
| --- | --- | --- | --- | --- | --- |
| a). To forget your worries? |  |  |  |  |  |
| b). Because it helps you when you feel depressed or irritated? |  |  |  |  |  |
| c). To cheer yourself up when you are in a bad mood? |  |  |  |  |  |
| d). To forget about your problems? |  |  |  |  |  |
| e). Because your friends pressurised you to do it? |  |  |  |  |  |
| f). Because you would like to belong to a certain circle of friends? |  |  |  |  |  |
| g). To be liked by others? |  |  |  |  |  |
| h). To not feel excluded? |  |  |  |  |  |
| i). Because it gives you a pleasant feeling? |  |  |  |  |  |
| j). Because it is exciting? |  |  |  |  |  |
| k). To experience a feeling of exaltation? (e.g. happiness) |  |  |  |  |  |
| l). Simply because it is fun? |  |  |  |  |  |
| m). To come into contact with others? |  |  |  |  |  |
| n). Because it is fun to be in contact with others? |  |  |  |  |  |
| o). To improve your contact with friends and acquaintances? |  |  |  |  |  |
| p). To share a special occasion with friends? |  |  |  |  |  |

- 1. **Please rate the extent to which you agree with the following statements, on a scale from *‘definitely disagree’* to ‘*definitely agree*’** *(select only one answer per statement)*

|  | Definitely Disagree | Strongly Disagree | Somewhat Disagree | Disagree | Agree | Somewhat Agree | Strongly Agree | Definitely Agree |
| --- | --- | --- | --- | --- | --- | --- | --- | --- |
| a). I have used social media to talk with others when I was feeling isolated |  |  |  |  |  |  |  |  |
| b). I would feel lost if I was unable to go on social media |  |  |  |  |  |  |  |  |
| c). I have difficulty controlling the amount of time I spend on social media |  |  |  |  |  |  |  |  |
| d). I prefer online social interaction over face-to-face communication |  |  |  |  |  |  |  |  |
| e). My social media use has made it difficult for me to manage my life |  |  |  |  |  |  |  |  |
| f). I have used social media to make myself feel better when I am down |  |  |  |  |  |  |  |  |
| g). When offline, I have a hard time trying to resist the urge to go on social media |  |  |  |  |  |  |  |  |
| h). My social media use has created problems for me in my life |  |  |  |  |  |  |  |  |
| i). I find it difficult to control my social media use |  |  |  |  |  |  |  |  |
| j). Online social interaction is more comfortable for me than face-to-face interaction |  |  |  |  |  |  |  |  |
| k). I have used social media to make myself feel better when I’ve felt upset |  |  |  |  |  |  |  |  |
| l). When I haven’t been on social media for some time, I become preoccupied with the thought of going on social media |  |  |  |  |  |  |  |  |
|  | Definitely Disagree | Strongly Disagree | Somewhat Disagree | Disagree | Agree | Somewhat Agree | Strongly Agree | Definitely Agree |
| m). I have missed social engagements or activities because of my social media use |  |  |  |  |  |  |  |  |
| n). I think obsessively about going on social media when I am offline |  |  |  |  |  |  |  |  |
| o). I prefer communicating with people online rather than face-to-face |  |  |  |  |  |  |  |  |

| SECTION 8: Smartphone and Social Media Use |
| --- |

This section is about how much you use your smartphone and social media during school and out of school. The following questions ask you to estimate how much time you are spending on your smartphone and social media.

- - 1. a). **Do you use your smartphone while you are in school?** *(please select one)*
- Yes
- No

b). **When do you use your smartphone while you are in school?**

- Anytime throughout the day (including during lessons)
- Only at breaks and lunchtimes
- Only after asking member of staff
- During lessons as directed by the teacher
- Only in designated zones
- Other *(please specify)* _______________________________________________

**23 a).** **On an average school day how much time do you spend on your phone during school hours (i.e. from when you enter the school grounds to when you leave the school grounds)?**

___________hours ________minutes (for example, “2 hours 40 minutes”)

**b).** **On an average school day how much time do you spend on social media during school hours (i.e. from when you enter the school grounds to when you leave the school grounds)?***Reminder: “social media” is any app on your smartphone that you use to interact with other people and that involves a profile to set up, for example; YouTube, TikTok, Instagram, Snapchat. Please do not include apps or platforms for entertainment/watching TV and gaming, such as; Netflix, Xbox chat and PlayStation hangout*

___________hours ________minutes (for example, “1 hour 40 minutes”)

- 1. **a).** **On an average school day, during the whole day (24 hours) how long do you spend on your phone? (i.e. from when you wake up until you go to sleep)?**

___________hours ________minutes (for example, “3 hours 40 minutes”)

**b).** **On an average school day, during the whole day (24 hours) how long do you spend on social media? (i.e. from when you wake up until you go to sleep)?**

___________hours ________minutes (for example, “2 hours 40 minutes”)

- 1. **a).** **On an average weekend day, during the whole day (24 hours) how long do you spend on your phone? (i.e. from when you wake up until you go to sleep)?**

___________hours ________minutes (for example, “2 hours 40 minutes”)

**b). On an average weekend day, during the whole day (24 hours) how long do you spend on social media? (i.e. from when you wake up until you go to sleep)?**

___________hours ________minutes (for example, “1 hour 40 minutes”)

- 1. **Please rate how much you agree with the following statements** *(select only one answer per statement)*

|  | Strongly Disagree | Disagree | Neutral | Agree | Strongly Agree |
| --- | --- | --- | --- | --- | --- |
| a). In my school, the majority of pupils **understand** the school’s rules on smartphone use during the school day |  |  |  |  |  |
| b). In my school, the majority of teachers **understand** the school’s rules for pupils on smartphone use during the school day |  |  |  |  |  |
| c). The majority of parents/carers of pupils in my school **understand** the school’s rules on smartphone use during the school day |  |  |  |  |  |
| d) In my school, the majority of pupils **are supportive of** the school’s rules on smartphone use during the school day |  |  |  |  |  |
| e) In my school, the majority of teachers **are supportive of** the school’s rules for pupils on smartphone use during the school day |  |  |  |  |  |
| f) The majority of parents/carers of pupils in my school **are supportive of** the school’s rules on smartphone use during the school day |  |  |  |  |  |
| g). In my school, the majority of pupils **follow** the school’s rules for smartphone use during the school day |  |  |  |  |  |
| h). In my school, the majority of teachers **ensure that pupils follow** the school’s rules on smartphone use during the school day |  |  |  |  |  |
| i). The majority of parents/carers of pupils in my school **ensure their children follow** the school’s rules on smartphone use during the school day |  |  |  |  |  |

| SECTION 9: Phone Task |
| --- |

This section is about how much you use your smartphone and social media during school and out of school. In order to answer the following questions, you will need to access data from your phone.

Please select which type of smartphone you have:

- Android: Please turn on your phone and turn on "Digital Wellbeing" in the "Settings" app to display the total screen time. Then, tap the dashboard and scroll down to display different apps used.
- IOS: Turn on the phone, open "Screen Time" in the "Settings" app, then click "See all activity.”

27 i). Can you see ‘Devices’ in the top right corner?

- Yes
- No (continue to question 27a)

ii) If Yes, Select ’Devices’ (in the top right corner) – is just your iPhone ticked?

- Yes
- No

iii) If ‘No’, what was ticked?

- Another of your devices (e.g. your iPad or iMac)
- A family member’s device (e.g. ‘Mum’s’ iPhone)
- All Devices

iv) If ‘All Devices’, how many devices does this include:

□ 1 □ 2 □ 3 □ 4 □ 5 □ Other (please specify)_________________

Make sure that only your iPhone is selected, not any other devices (e.g. iPad) nor ‘all devices’.

**27 a).** Scroll down to see all the different apps used. Use your phone to write down the answers to the following;

**On the last day you were at school, what was your total screen time on your smartphone *(for example, if today was Wednesday, your last day at school would be Tuesday)*?**

___________hours ________minutes (for example, “2 hours 40 minutes”)

**b). On the last day you were at school, how much time did you spend on different social media apps *(for example, if today was Wednesday, your last day at school would be Tuesday)*?**

You will need to scroll down on your phone and list the different time spent on social media

- Snapchat: ___________hours ________minutes
- TikTok**: ___________**hours ________minutes
- Instagram: ___________hours ________minutes
- WhatsApp ___________hours ________minutes
- Facebook: ___________hours ________minutes
- Twitter: ___________hours ________minutes
- YouTube: ___________hours ________minutes
- Reddit: ___________hours ________minutes
- Discord: ___________hours ________minutes
- Pinterest: ___________hours ________minutes
- Tumblr: ___________hours ________minutes
- Lobby: ___________hours ________minutes
- Yubo: ___________hours ________minutes
- Twitch: ___________hours ________minutes
- Other: (Please specify) Social media ………. __________hours _______minutes
- Other: (Please specify) Social media ………. ___________hours ________minutes
- Other: (Please specify) Social media ………. ___________hours ________minutes
- Other: (Please specify) Social media ………. ___________hours ________minutes
- Other: (Please specify) Social media ………. ___________hours ________minutes
- Other: (Please specify) Social media ………. ___________hours ________minutes

**c). Select a typical weekend day last weekend, and report on your total screen time on your phone *(for example, Saturday or Sunday)*?**

**Please Select Saturday or Sunday:**

- Saturday
- Sunday

___________hours ________minutes (for example, “2 hours 40 minutes”)

You will need to scroll down on your phone and list the different time spent on social media

- Snapchat: ___________hours ________minutes
- TikTok**: ___________**hours ________minutes
- Instagram: ___________hours ________minutes
- WhatsApp ___________hours ________minutes
- Facebook: ___________hours ________minutes
- Twitter: ___________hours ________minutes
- YouTube: ___________hours ________minutes
- Reddit: ___________hours ________minutes
- Discord: ___________hours ________minutes
- Pinterest: ___________hours ________minutes
- Tumblr: ___________hours ________minutes
- Lobby: ___________hours ________minutes
- Yubo: ___________hours ________minutes
- Twitch: ___________hours ________minutes
- Other: (Please specify) Social media ………. ___________hours ________minutes
- Other: (Please specify) Social media ………. ___________hours ________minutes
- Other: (Please specify) Social media ………. ___________hours ________minutes
- Other: (Please specify) Social media ………. ___________hours ________minutes
- Other: (Please specify) Social media ………. ___________hours ________minutes
- Other: (Please specify) Social media ………. ___________hours ________minutes

| END |
| --- |

**Would you like to be contacted in the future about taking part in further research with the University of Birmingham?**

- Yes
- No

**If yes, please insert email address__________________________________**

**You have reached the end of the survey.**

**Thank you very much for taking part in this study.**

If, after finishing the survey, you feel you would like some further information on wellbeing support, the following resources may be useful to you:

- [www.kooth.com](http://www.kooth.com)
- [www.breathe-edu.co.uk/local-services](http://www.breathe-edu.co.uk/local-services)

If you feel uncomfortable or upset for any reason please tell a teacher in your school or your school wellbeing officer or your parent/carer. The Young Minds Charity also provides some practical advice to helping you find support, and this can be accessed here: <https://www.youngminds.org.uk/young-person/> You can also access support by calling Childline on 08001111

Now that you have completed the survey, if you would like to, you can do another activity to help with wellbeing and relaxation. Please click on the link and follow the instructions: <https://bit.ly/38bita1>

If you have any questions, please do not hesitate to contact the research team on 0121 414 3158 or email smartstudy@contacts.bham.ac.uk

For further information about the study or updates on the study, please visit our website at birmingham.ac.uk/smart-schools

# STROBE Checklist

|  | Item No. | Recommendation | Page  No. | Relevant text from manuscript |
| --- | --- | --- | --- | --- |
| **Title and abstract** | 1 | (*a*) Indicate the study’s design with a commonly used term in the title or the abstract | 1 | Cross Sectional Observational Study. |
|  |  | (*b*) Provide in the abstract an informative and balanced summary of what was done and what was found | 2 | Background: Poor mental health in adolescents can negatively affect sleep, physical activity and academic performance, and is attributed by some to increasing mobile phone use. Many countries have introduced policies to restrict phone use in schools to improve health and educational outcomes. The SMART Schools study evaluated the impact of school phone policies by comparing outcomes in adolescents who attended schools that restrict and permit phone use.  Methods: We conducted a cross-sectional observational study with adolescents from 30 English secondary schools, comprising 20 with restrictive (recreational phone use is not permitted) and 10 with permissive (recreational phone use is permitted) policies. The primary outcome was mental wellbeing (assessed using Warwick-Edinburgh Mental Well-Being Scale (WEMWBS). Secondary outcomes included smartphone and social media time. Mixed effects linear regression models were used to explore associations between school phone policy and participant outcomes, and between phone and social media use time and participant outcomes. Study registration: ISRCTN77948572.  Findings: We recruited 1227 participants (age 12-15) across 30 schools. Mean WEMWBS score was 47 (SD=9) with no evidence of a difference between groups (adjusted mean difference -0·48, 95% CI -2·05 to 1·06, p=0·62). Adolescents attending schools with restrictive, compared to permissive policies had lower phone (adjusted mean difference -0·67 hours, 95% CI -0·92 to -0·43, p=0·00024) and social media time (adjusted mean difference -0·54 hours, 95% CI -0·74 to -0·36, p=0·00018) during school time, but there was no evidence for differences when comparing usage time on weekdays or weekends.  Interpretation: There is no evidence that restrictive school policies are associated with overall phone and social media use or better mental wellbeing in adolescents. The findings do not provide evidence to support the use of school policies that prohibit phone use during the school day in their current form, and indicate that these policies require further development. |
| Introduction | | | |  |
| Background/rationale | 2 | Explain the scientific background and rationale for the investigation being reported | 3 | In the last few years, there has been a growing international trend for the use of phones to be prohibited in schools.^25^ The UN reported that one in four countries (including France, Israel, and Turkey, as well as regions of Canada and Australia) have introduced laws that mandate public schools to prohibit phone use during the school day.^25^ Other countries, such as the UK, provide non-statutory guidance recommending prohibiting phones in schools, where prohibiting phone use is left to the school’s discretion.^26^ However, prior to recent legislation and guidance, many schools had opted to devise their own policies that restrict phone use during the school day.^25-27^ Overall, restrictive school phone policies are based on popular assumptions that prohibiting phone use in schools will improve mental health and wellbeing, educational attainment and reduce problematic use and levels of disruptive behaviour.^25-27^ There is some evidence indicating that restrictive school phone policies in the UK lower adolescents’ uses of electronic communication devices (including mobile phones).^28^ However, there are currently no published peer-reviewed studies reporting on the association between school phone policies, adolescent phone/media use behaviours and mental health, wellbeing and other related outcomes (e.g., sleep, physical activity, educational attainment and behaviour).^26,27^ |
| Objectives | 3 | State specific objectives, including any prespecified hypotheses | 3-4 | Based on the available evidence of associations between phone use and mental health and wellbeing, we hypothesised that school policies that restrict the daytime use of phones would lower the overall time adolescents spend on phones/social media and improve adolescent mental wellbeing, possibly operating through improving related outcomes (e.g., physical activity, sleep, academic performance, and classroom behaviour). The logic model in Supplementary Figure 1 presents these processes. The research questions for this study were:   1. In schools that do not permit smartphone use compared with schools that permit smartphone use: 2. Is there a difference in mental wellbeing, anxiety and depression, sleep duration, time spent in physical activity, classroom disruptive behaviour, attainment and prevalence of problematic use? 3. Is there a difference in smartphone and social media use and duration of use within school, over a 24hr period and across 7 days and is there a difference in motives for phone/media use? 4. Is there an association between smartphone and social media time and mental wellbeing, anxiety, depression, sleep duration, time spent in physical activity, classroom disruptive behaviour, attainment, and prevalence of problematic use? |
| Methods | | | |  |
| Study design | 4 | Present key elements of study design early in the paper | 4 | The SMART Schools study was a multi-method cross-sectional observational study, designed to evaluate the impact of school phone policies by comparing mental health and wellbeing, sleep, physical activity, and education outcomes in adolescents who attended schools that restrict and permit phone use during the school day. In this paper we focus on the findings from the quantitative observational element of the study. |
| Setting | 5 | Describe the setting, locations, and relevant dates, including periods of recruitment, exposure, follow-up, and data collection | Separate file  4 | Study profile Figure 1  School recruitment commenced in September 2022 and was completed in March 2023 |
| Participants | 6 | (*a*) *Cohort study*—Give the eligibility criteria, and the sources and methods of selection of participants. Describe methods of follow-up  *Case-control study*—Give the eligibility criteria, and the sources and methods of case ascertainment and control selection. Give the rationale for the choice of cases and controls  *Cross-sectional study*—Give the eligibility criteria, and the sources and methods of selection of participants | 4-5 | School recruitment commenced in September 2022 and was completed in March 2023. The recruitment process is outlined in Figure 1. The final sampling frame included 1341 state funded mainstream secondary schools (age 11-19) in England located within a 100-mile radius of the recruiting centre. Secondary schools were included as most adolescents in England own a smartphone by age 11.^9^ Schools other than state-funded mainstream schools (special schools, pupil referral units and independent schools) were excluded because it was expected that there would be additional influences on mental wellbeing.^27^ Schools that did not have an accessible smartphone policy and/or had different phone policies for different year groups in mainstream education were excluded. Informed by our patient and public involvement (PPI) activities, school website and policy analysis,^26,27^ we classified school phone policies as either restrictive (intervention) or permissive (comparator). In restrictive schools, phones were not allowed to be used during the school day for recreational purposes, and were required to be kept off inside bags, stored in lockers, kept in a pouch, handed into the school reception or phones were not allowed onto the school premises altogether (see Table 1).^26^ In permissive schools, phones were permitted to be used at any time or at certain times (e.g., breaks/lunch) and/or in certain zones (e.g., outside) (see Table 1).^26^ |
|  |  | (*b*) *Cohort study*—For matched studies, give matching criteria and number of exposed and unexposed  *Case-control study*—For matched studies, give matching criteria and the number of controls per case |  |  |
| Variables | 7 | Clearly define all outcomes, exposures, predictors, potential confounders, and effect modifiers. Give diagnostic criteria, if applicable | Panel | Table 1 in the Supplementary File, and notes on Adjustment Variables in the Supplementary File. |
| Data sources/ measurement | 8* | For each variable of interest, give sources of data and details of methods of assessment (measurement). Describe comparability of assessment methods if there is more than one group | Panel | Table 1 in the Supplementary File, and notes on Adjustment Variables in the Supplementary File. |
| Bias | 9 | Describe any efforts to address potential sources of bias | 5 | There were no ineligibility criteria for pupils, and all pupils within the participating classes were invited to take part, thus reducing any potential of selection bias. |
| Study size | 10 | Explain how the study size was arrived at | 6 | To account for the imbalance of schools in our sampling frame that had restrictive (n =1245) and permissive policies (n= 96), we recruited schools using a 2:1 ratio. The primary outcome of mental wellbeing was measured using WEMWBS (score range =14-70). To detect a mean difference in score of 3 (considered the minimum clinically important difference^36^ between the two school groups), assuming a SD of 6·8^37^ and an ICC of 0·1 (a conservative estimate)^38^, with 90% power and 5% significance, we required 20 schools in the restrictive and 10 schools in the permissive phone policy groups, with an average cluster size of 39 pupils (1170 pupil participants in total; 780 in the restrictive, and 390 in the permissive policy groups). |
| Quantitative variables | 11 | Explain how quantitative variables were handled in the analyses. If applicable, describe which groupings were chosen and why | Separate file | Table 1 in the Supplementary File |
| Statistical methods | 12 | (*a*) Describe all statistical methods, including those used to control for confounding | 6-7 | All analysis was conducted using R Statistics (version 4.1.2) and R Studio. For the primary outcome of mental wellbeing, a mean was calculated from the two measures for each individual and its association with phone policy type was investigated using mixed effects linear regression which included year group (year 8 or year 10) and school (30 schools) as random effects variables. School- and pupil-level covariates were included as fixed effects adjustment variables (see Supplementary File). The adjustment variables were identified based on the available literature on the key factors that may influence mental wellbeing and/or phone/media use.  The association between phone policy type and the secondary outcomes were explored using mixed effects linear regression, except for attainment scores, where mixed effects logistic regression was used. All regression models included the same random and fixed effects covariates except for the attainment mixed effects logistic regression model which included year group as a fixed, instead of a random effect to allow convergence. For all schools in the study, we investigated the association between smartphone and social media time and the primary and secondary outcomes using mixed effect models, including the same adjustment variables (Supplementary File). |
|  |  | (*b*) Describe any methods used to examine subgroups and interactions | 7 | Based on the literature on adolescent mental wellbeing, we identified four subgroup effects that were of interest: deprivation, sex, ethnicity, and year group. We separately introduced interactions between pupil sex, ethnicity, year group, school IDACI, and school smartphone policy into the models. We performed a sensitivity analysis using only the first WEMWBS score provided by participants to explore the potential influence of the experience of the first data collection point on the second completion of the WEMWBS questions. Self-reported phone use was used for our primary analysis due to concerns over the accuracy of the SR phone data (Supplementary File). There was a high proportion of missing data due to input errors and the way the phone apps may have been miscounting social time, resulting in nearly a third of participants reporting higher social media times than phone times. These issues were comparable across permissive and restrictive schools (see Supplementary File). We found that the self-reported and SR phone data measures had a strong correlation (Supplementary File). Only self-reported phone and social media use is reported from this point forwards.  We modelled relationships between phone and social media time and other outcomes as linear based on our exploratory data analysis (Supplementary File), however we explored non-linearity in these relationships by re-running the models using a log(x + 1) transformation for the phone and social media duration variables (models are not reported as there were no meaningful changes in interpretation of the associations). |
|  |  | (*c*) Explain how missing data were addressed | Separate file  6 | Table 1 in the Supplementary File  Missing data was not imputed, other than for the following: where a pupil had missing IMD (Index of Multiple Deprivation) data (n=120), the median IMD rank for pupils in their school was used; where a pupil had a missing Date of Birth (n=4), the median age for their year group in their school was used; missing responses on validated outcome measures were addressed as outlined in the Supplementary File |
|  |  | (*d*) *Cohort study*—If applicable, explain how loss to follow-up was addressed  *Case-control study*—If applicable, explain how matching of cases and controls was addressed  *Cross-sectional study*—If applicable, describe analytical methods taking account of sampling strategy | N/A  N/A  N/A | N/A  N/A  N/A |
|  |  | (*e*) Describe any sensitivity analyses | 7 | We performed a sensitivity analysis using only the first WEMWBS score provided by participants to explore the potential influence of the experience of the first data collection point on the second completion of the WEMWBS questions.  Additionally, we performed a sensitivity analysis with a small sample using only data from pupils in schools where restrictive phone policies required phones to be inaccessible to pupils (n=4), to explore whether policies with greater levels of restrictions on pupils’ access to phones influenced outcomes. We performed a sensitivity analysis with weekend smartphone use as the control variable to account for the relationship between in-school and leisure time phone use. |
| Results |  |  |  |  |
| Participants | 13* | (a) Report numbers of individuals at each stage of study—eg numbers potentially eligible, examined for eligibility, confirmed eligible, included in the study, completing follow-up, and analysed | Figure 1  7 | Study profile figure  Of the recruited pupil participants, 1223 (99·67%) provided data on mental wellbeing (primary outcome); 817 (99·63%) in the restrictive schools (intervention) and 406 (99·75%) in permissive schools (comparator) (Figure 1). |
|  |  | (b) Give reasons for non-participation at each stage | Figure 1 | Study profile figure |
|  |  | (c) Consider use of a flow diagram | Figure 1 | Study profile figure |
| Descriptive data | 14* | (a) Give characteristics of study participants (eg demographic, clinical, social) and information on exposures and potential confounders | Table 1 | Table 7 Supplementary Appendix |
|  |  | (b) Indicate number of participants with missing data for each variable of interest | Table 2 | Table 2  Table 7 Supplementary Appendix |
|  |  | © *Cohort study*—Summarise follow-up time (eg, average and total amount) | N/A | N/A |
| Outcome data | 15* | *Cohort study*—Report numbers of outcome events or summary measures over time | Table 2 | Table 2  Table 7 Supplementary Appendix |
|  |  | *Case-control study—*Report numbers in each exposure category, or summary measures of exposure | N/A | N/A |
|  |  | *Cross-sectional study—*Report numbers of outcome events or summary measures | N/A | N/A |
| Main results | 16 | (*a*) Give unadjusted estimates and, if applicable, confounder-adjusted estimates and their precision (eg, 95% confidence interval). Make clear which confounders were adjusted for and why they were included | Figure 2  Supplementary Appendix | Figure 2  Table 3  Table 4  Table 5  Supplementary Appendix |
|  |  | (*b*) Report category boundaries when continuous variables were categorized | N/A | N/A |
|  |  | (*c*) If relevant, consider translating estimates of relative risk into absolute risk for a meaningful time period | N/A | N/A |
| Other analyses | 17 | Report other analyses done—eg analyses of subgroups and interactions, and sensitivity analyses | 8  Supplementary Appendix | No statistically significant interactions were observed for mental wellbeing across sex, year group, ethnicity and IDACI (see Supplementary File). In the sensitivity analysis using only the first WEMWBS measure there was no significant difference in mental wellbeing between the two school groups (see Supplementary File).  There was some variability in the distribution of the time spent on smartphones and social media during school hours in both permissive and restrictive schools (Figure 2). In the sensitivity analysis using only restrictive schools where phones were inaccessible to pupils, there were no significant differences with permissive schools across all outcomes (Supplementary File).  In the sensitivity analysis controlling for weekend smartphone time, no significant differences were found for all outcomes (see Supplementary File). |
| Discussion |  |  |  |  |
| Key results | 18 | Summarise key results with reference to study objectives | 9 | There was no significant difference in adolescent mental wellbeing between pupils attending schools that permitted phone use compared to pupils attending schools that restricted phone use. However, there were negative associations between increasing time spent on phones/social media and lower mental wellbeing. Similarly, we observed no significant differences in anxiety, depression, problematic social media use, sleep, physical activity, attainment, and disruptive behaviour when comparing adolescents exposed to restrictive or permissive school phone policies, but we did observe significant negative associations between these outcomes and increasing phone and social media time. This study therefore provides further evidence of the adverse consequences from increased smartphone and social media use, and that lowering phone and social media use is important. |
| Limitations | 19 | Discuss limitations of the study, taking into account sources of potential bias or imprecision. Discuss both direction and magnitude of any potential bias | 10-11 | This study used a cross-sectional observational approach to explore the potential effects of school phone policies in a large nationally representative cohort, using regression analysis to control for possible confounding factors. The recruited schools differed in several ways from those in the sampling frame (e.g., academy status, selective, sixth form, faith), and given the school level response rates, this may have been a source of selection bias impacting on the generalizability of the findings. There were also some differences in the permissive and restrictive sample of schools (e.g., a greater proportion of selective, single sex and secular schools in the permissive school category), and this reflected the differences in the permissive and restrictive schools in the sampling frame. School characteristics were controlled for in the analyses. Overall, therefore, despite some sample limitations, the results from this study are likely to be applicable across the UK. A limitation is that the study design is cross sectional which makes it difficult to draw conclusions about causality and reverse causality cannot be ruled out. Furthermore, there is a risk of selection bias and unmeasured confounding in this study. For example, other school characteristics, such as differing school policies may impact on the outcomes, possibly having a confounding effect.  The self-reported data on phone and social media use duration collected in this study may have introduced a bias; pupils in schools with restrictive phone policies may have been more likely to under-report their in-school phone use, compared with those in schools with permissive policies. If this were the case, the difference in in-school use between the two groups that we report would be an over-estimate. Data were collected from adolescent phones on screen time and social media use; however, we were unable to include these measures in our analyses, due to concerns related to the accuracy of adolescents self-reporting phone and social media data from apps, as well as a high proportion of implausible data. Future studies should explore ways to access for research purposes the data that are available on adolescents’ phones. Our questionnaire did not ask adolescents if they did not have a smartphone, and future studies should explore how outcomes in non-users compare. Gender identity groups that differ from sex registered at birth should be explored further, as in this study the group was too small to incorporate as an adjustment variable to say anything in confidence. |
| Interpretation | 20 | Give a cautious overall interpretation of results considering objectives, limitations, multiplicity of analyses, results from similar studies, and other relevant evidence | 11 | In conclusion, school phone policies that restrict the daytime use of phones lower the time adolescents spend on phones/social during their time in school but are not associated with an overall reduction in the time adolescents spend on phones and social media. In addition, there is no evidence to support that restrictive school phone policies, in their current forms, have a beneficial effect on adolescents’ mental health and wellbeing or related outcomes, indicating that the intentions of these policies to improve adolescent health, wellbeing and educational engagement are not realised. Our data suggest that interventions to reduce phone/social media time to positively influence adolescent mental wellbeing are plausible, but that both in-school and outside of school use should be considered in tandem. Preventative efforts should also consider how other behaviours that influence wellbeing are influenced by increased phone/media use, such as sleep, physical activity, attainment, classroom behaviour, and problematic use. In the design of new guidelines and interventions, phone and media use could be approached as part of a ‘compositional whole’, whereby phone/media time guidance focuses on obtaining the ‘right balance’ between time spent on devices and other daily lifestyle behaviours. This approach does not necessarily preclude restrictive school mobile phone policies, but these policies would be linked with a wider holistic approach to adolescent mobile phone and social media use. This is comparable to the 24-hour model taken in the study of movement behaviours, and in the design of physical activity guidelines. |
| Generalisability | 21 | Discuss the generalisability (external validity) of the study results | 10 | This study used an observational approach to explore the potential effects of school phone policies in a large nationally representative cohort, using regression analysis to control for possible confounding factors. |
| Other information |  |  |  |  |
| Funding | 22 | Give the source of funding and the role of the funders for the present study and, if applicable, for the original study on which the present article is based | 7 | This study is funded by the National Institute for Health Research (NIHR). The funder of the study had no role in study design, data collection, data analysis, data interpretation, or writing of the report, or the decision to submit the Article for publication. |

*Give information separately for cases and controls in case-control studies and, if applicable, for exposed and unexposed groups in cohort and cross-sectional studies.

**Note:** An Explanation and Elaboration article discusses each checklist item and gives methodological background and published examples of transparent reporting. The STROBE checklist is best used in conjunction with this article (freely available on the Web sites of PLoS Medicine at http://www.plosmedicine.org/, Annals of Internal Medicine at http://www.annals.org/, and Epidemiology at http://www.epidem.com/). Information on the STROBE Initiative is available at www.strobe-statement.org.
